# Topological Singularities and Edge‐State Coupling Enable Robust on‐Chip Slow Light

**DOI:** 10.1002/advs.202507226

**Published:** 2025-07-02

**Authors:** Yuqian Wang, Shengyu Hu, Zhiwei Guo, Jie Jiang, Yaping Yang, Cuicui Lu, Hong Chen

**Affiliations:** ^1^ MOE Key Laboratory of Advanced Micro‐structured Materials School of Physics Sciences and Engineering Tongji University Shanghai 200092 China; ^2^ Key Laboratory of Advanced Optoelectronic Quantum Architecture and Measurements of Ministry of Education Beijing Key Laboratory of Nanophotonics and Ultrafine Optoelectronic Systems School of Physics Beijing Institute of Technology Beijing 100081 China

**Keywords:** edge state, on‐chip, quantum interference, slow light, topological singularity

## Abstract

Quantum interference (QI) between multiple excitation pathways can manipulate high‐quality optical responses, like slow light, highly‐sensitive sensors, and ultrafast switches. However, the enhanced light‐matter interactions and reduced group velocity tend to arouse high sensitivity to the environment, challenging fabrication and operation in practical applications. In this work, the first experimental demonstration of topological QI‐like effect is reported in a 1D on‐chip system, where two different topological mechanisms are induced to provide robustness for slow light. Topological charges rooted in the parameter space can offer immunity to parameter deviations, such as coupling strengths, and topological edge states rooted in the momentum space can manifest robustness against structural disturbances, like width disorders and bending deformations. By incorporating bright and dark edge states into a composite waveguide, the electromagnetically induced transparency (EIT) window is observed and switching between slow light and fast light is demonstrated by measuring the transmission and group delays. The findings provide an extensible platform for exploring novel QI and topological physics, and pave the avenue for developing robust on‐chip devices.

## Graphical abstract

This work demonstrates the first experimental observation of a topological quantum‐interference‐like effect in a 1D on‐chip system, utilizing two topological mechanisms for robust slow light. Topological charges ensure immunity to parameter deviations, while edge states provide robustness against disturbances. The system exhibits topological electromagnetically induced transparency and switching between slow and fast light.

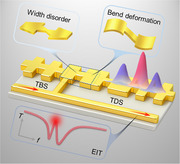

## 1 Introduction

Quantum interference (QI) effect originates from coherent interactions between multiple excitation pathways.^[^ ^1^, ^2^ ^]^ Recently, photon degrees of freedom (DOF), including phase,^[^ ^3^ ^]^ polarization,^[^ ^4^ ^]^ and orbital angular momentum^[^ ^5^ ^]^ have been induced and leveraged to manipulate high‐quality optical responses, arousing potential applications in slow light (SL),^[^ ^6^, ^7^ ^]^ highly‐sensitive sensors,^[^ ^8^, ^9^ ^]^ and ultrafast switches.^[^ ^10^, ^11^ ^]^ Especially for SL, the enhanced light‐matter interactions and reduced group velocity imply keen sensitivity to structural parameters, which poses challenges for fabrication and operational precision. Therefore, achieving robust, coherent interactions is crucial for the reliable implementation of related devices.

On the other hand, band topological properties have been transferred from the field of condensed matter to quantum optics, providing a novel DOF with robustness for controlling the flow of photons.^[^ ^12^, ^13^, ^14^, ^15^, ^16^, ^17^ ^]^ Recently, topological SL has garnered significant attention based on flat bands. Wherein the topological properties mainly originate from quantum valley‐Hall^[^ ^18^, ^19^ ^]^ or anomalous‐Hall effects,^[^ ^20^, ^21^, ^22^ ^]^ which tend to require 2D settings. Take the anomalous Hall effect as an example, a 2D photonic Chern insulator possesses chiral edge states, which are protected by the nontrivial Chern number. When coupling to several resonator modes, the hybridized edge state not only inherits the topological property immune to disorders, but also completes multiple windings around the Brillouin zone to reduce the group velocity.^[^ ^22^ ^]^ Similar designs tend to require external magnetic fields to break time‐reversal symmetry.

To overcome the fundamental challenges of miniaturizing and integrating topological photonic devices into practical schemes, on‐chip and integrated topological photonics have attracted extensive attention.^[^ ^23^, ^24^, ^25^, ^26^, ^27^, ^28^, ^29^ ^]^ In this work, we employ a 1D waveguide‐based on‐chip structure in a classical transmission line platform to experimentally demonstrate the emergence of topological QI‐like effect owing to near‐field coupling between edge states. In this system, two kinds of topology concepts contribute to enhancing and stabilizing the strong SL and fast light (FL). On the one hand, topological charges exhibit immunity to parameter deviations. On the other hand, topological edge states demonstrate robustness against structural disturbances, like width disorders and bending deformations, which occur in fabrications and operations. Moreover, the proposed QI‐like platform features a planar structure that is easier to integrate and exhibits lower losses. The field of topological physics facilitates the study of the coherent interactions of light and may promote novel on‐chip wave‐functional applications with topological protection.

## 2 Results

**Figure 1:**
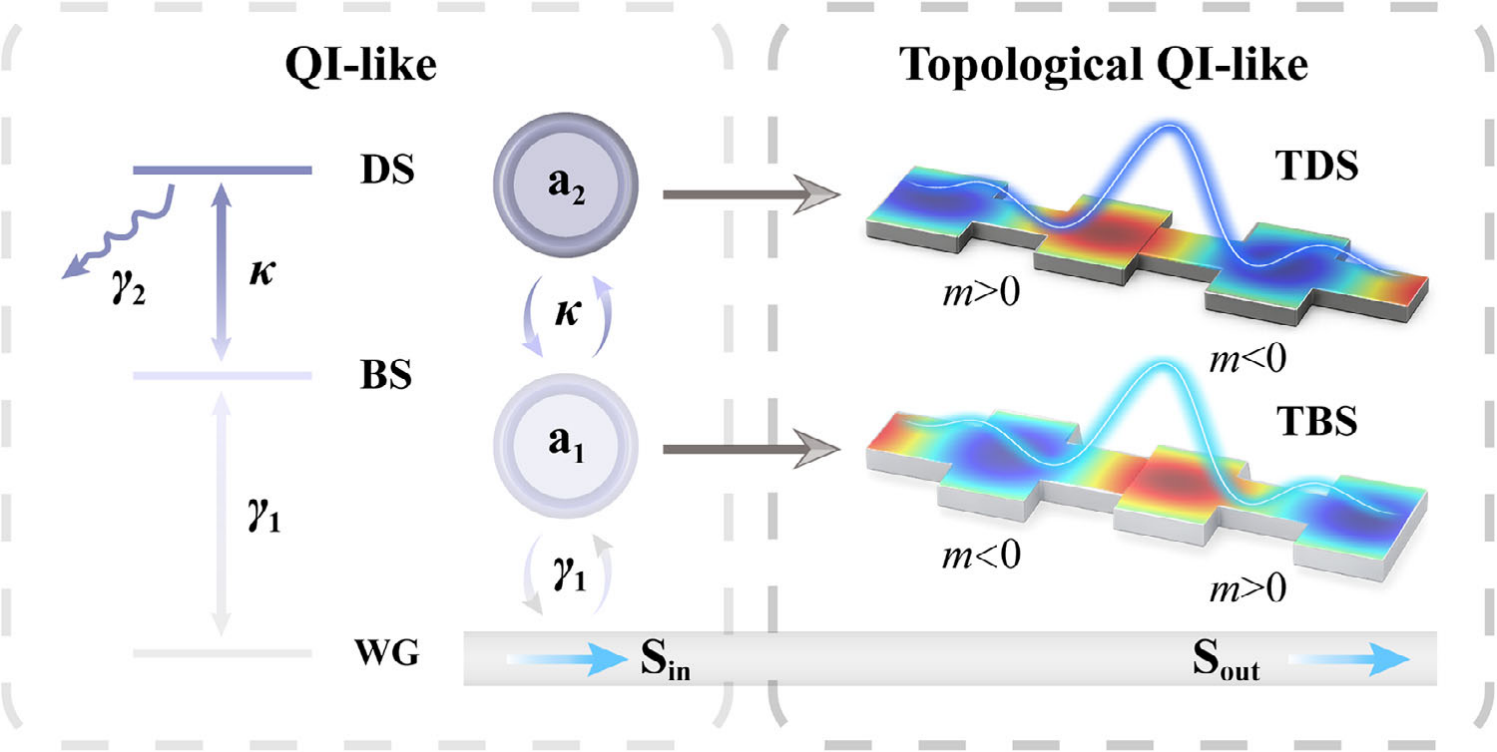
Topological QI‐like effect established by the coupled TBS and TDS. Schematic of the waveguide‐based structure. The TBS (in bright blue) and TDS (in dark blue) are formed at the interfaces between two topologically distinguished waveguides (WG) with different effective mass *m*.

The schematic of the waveguide‐based topological QI‐like structure is shown in **Figure** **1**. As an example, two topological edge states (Figures S1–S4, Supporting Information), originating from the heterojunction of two topologically distinguished waveguides with different topological invariants,^[^ ^30^, ^31^, ^32^, ^33^ ^]^ play the role of the basic atoms. When a continuous harmonic wave *S_in_* = *s_in_* *e* ^*i* *ω* *t*^ is at an angular frequency *ω* = 2π*f* incident to the system, the lower atom (in bright blue) can be excited directly by the incoming wave *S_in_*, serving as the topological bright state (TBS), while the upper atom (in dark blue) must be excited via the TBS, corresponding to the topological dark state (TDS).

Considering the near‐field coupling *κ* between two atoms (Figures S5–S7, Supporting Information), the dynamic equations can be written using a temporal coupled‐mode theory as^[^ ^34^ ^]^

(1) $$\begin{array}{c} \frac{1}{2\pi }\frac{\partial a_{1}}{\partial t}=\left(if_{1}-\gamma_{1}\right)a_{1}+i\kappa a_{2}+i\sqrt{\gamma_{1r}}S_{in}\\ \frac{1}{2\pi }\frac{\partial a_{2}}{\partial t}=\left(if_{2}-\gamma_{2}\right)a_{2}+i\kappa a_{1} \end{array}$$

where the resonance frequency and damping of TBS (TDS) are *f*_1_ (*f*_2_) and *γ*_1_ = *γ*_1*d*_  + *γ*_1*r*_ (*γ*_2_ = *γ*_2*d*_ ), respectively. In addition, further analysis of parity‐time symmetry (PT‐symmetry) and broken PT‐symmetry in the coupled edge state non‐Hermitian system is provided in the supplementary material (Figures S8–S10, Supporting Information).

**Figure 2:**
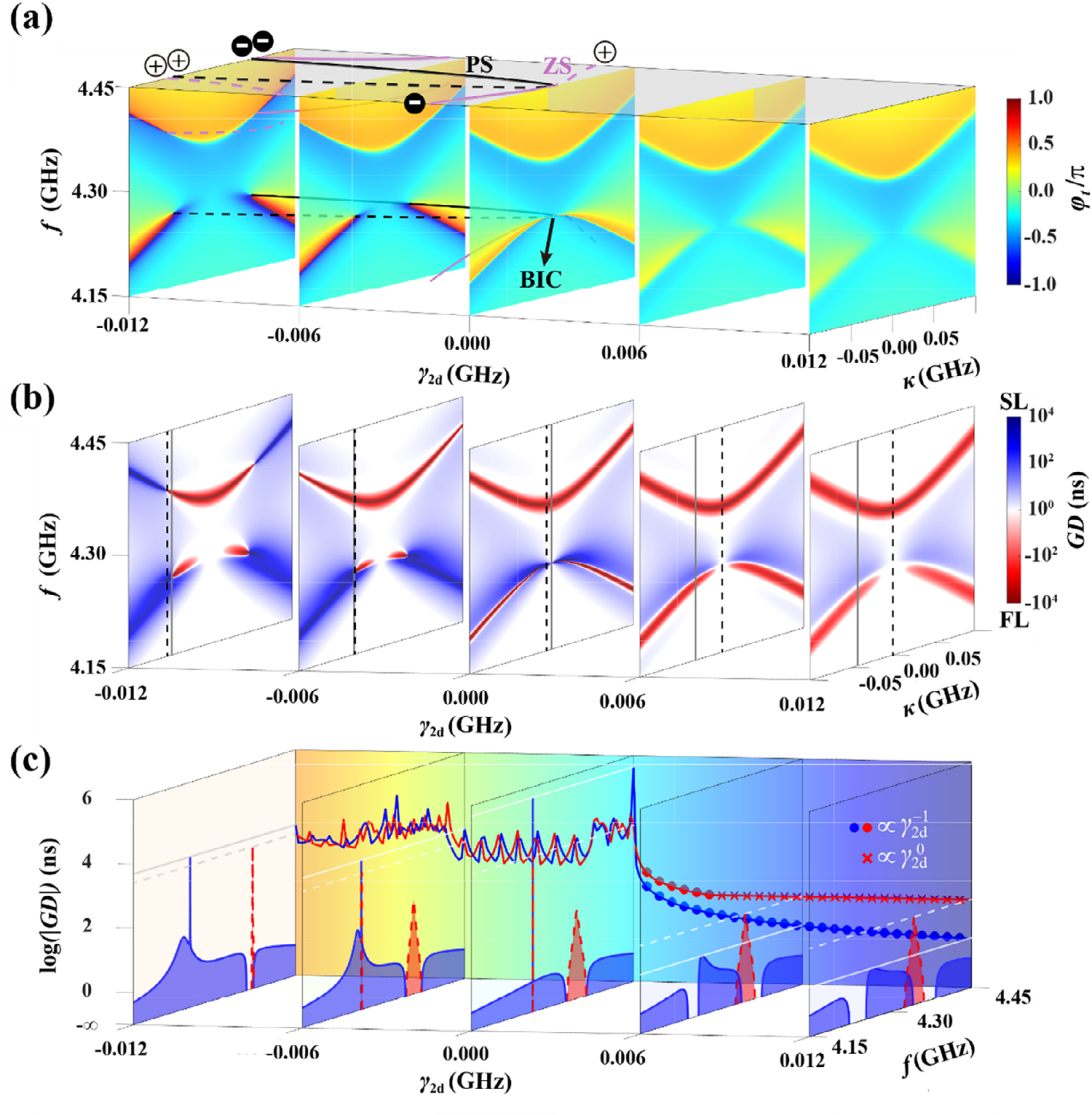
*GD* modulation near topological singularities. a) Susceptibility slices with different *γ*_2*d*_, and the trajectories of singularities are projected to the top plane. b) Related *GD* slices, where the strongest SL and FL are located on the solid and dashed lines, respectively. c) The continuous evolution of the strongest SL (blue) and FL (red) on the back plane. On the slices, the discrete spectra of *GD* related to the solid and dashed lines in (b). For clarity, only the interested parts (the blue parts of solid lines and the red parts of dashed lines) are plotted. Here *f*_1_ =  4.344 GHz, *f*_2_ =  4.264 GHz, *γ*_1*d*_ =  0.003 GHz, and *γ*_1*r*_ =  0.11 GHz are fixed.

More specifically, the TBS has both the dissipative loss *γ*_1*d*_ and the radiative loss *γ*_1*r*_, while the TDS only has the dissipative loss *γ*_2*d*_. And the transmission *T* = |*t*|^2^ can be obtained with $t=1-\frac{\gamma_{1r}\left[i\left(f-f_{2}\right)+\gamma_{2d}\right]}{\left[i\left(f-f_{1}\right)+\gamma_{1}\right]\left[i\left(f-f_{2}\right)+\gamma_{2d}\right]+\kappa^{2}}$. As shown in **Figure** **2**a (*γ*_2*d*_ = −0.001 GHz is shown in Figure S11, Supporting Information), the peculiar case *t* = 0 or t→∞ can arouse ill‐defined susceptibilities $\varphi_{t}=-i\log\left(\frac{t}{\left|t\right|}\right)$, namely the zero singularity (ZS, marked by the violet lines) at

(2) $$\begin{array}{c} f=\left(f_{1}\gamma_{2d}+f_{2}\gamma_{1d}\right)/\left(\gamma_{1d}+\gamma_{2d}\right)\\ \kappa =\pm \sqrt{-\gamma_{1d}\gamma_{2d}\left[1+{\left(f_{1}-f_{2}\right)}^{2}/{\left(\gamma_{1d}+\gamma_{2d}\right)}^{2}\right]} \end{array}$$

or the pole singularity (PS, marked by the black lines) at

(3) $$\begin{array}{c} f=\left(f_{1}\gamma_{2d}+f_{2}\gamma_{1}\right)/\left(\gamma_{1}+\gamma_{2d}\right)\\ \kappa =\pm \sqrt{-\gamma_{1}\gamma_{2d}\left[1+{\left(f_{1}-f_{2}\right)}^{2}/{\left(\gamma_{1}+\gamma_{2d}\right)}^{2}\right]} \end{array}$$

These singularities are characterized by the accumulated ± 2π phase around them in *f* − *κ* space, with the topological charge $\nu =\int_{C}\frac{d\varphi_{t}}{2\pi }=\pm 1$ along an anticlockwise closed loop.^[^ ^35^, ^36^ ^]^ Near the singularities shown in Figure 2b, the sensitive susceptibility can engender a dramatic transition between SL (*GD* > 0) and FL (*GD* < 0),^[^ ^37^ ^]^ where *GD*  =  ∂φ_*t*_/∂ω is the group delay.^[^ ^38^ ^]^ Noteworthy, the singularities are robust against parameter deviations before annihilation with each other. We modulate γ_2*d*_ < 0 and extract the maximum and minimum of *GD* on each slice, reflecting the strongest SL and FL, which oscillate due to the sensitive *φ*_*t*_ but sustain a high value near $GD∼10^{4}$ in the back plane of Figure 2c.

To further explore the spectral characteristics, the effective Hamiltonian for the open situation can be written as:^[^ ^39^ ^]^

(4) $$H=\left(\begin{array}{cc} f_{1}-i\gamma_{1} & \kappa \\ \kappa & f_{2}+i\gamma_{2} \end{array}\right)$$

The eigenvalues are $\alpha_{\pm }=\frac{1}{2}\left(f_{+}+i\gamma_{+}\right)\pm \frac{1}{2}\sqrt{4\kappa^{2}+{\left(f_{-}+i\gamma_{-}\right)}^{2}}$ with *f*_±_ = *f*_1_  ± *f*_2_ and *γ*_±_ =   − *γ*_1_ ± *γ*_2_. For *κ* = 0 and *γ*_2*d*_ = 0, the pure real *α*_−_ indicates a bound state in the continuum (BIC),^[^ ^40^, ^41^ ^]^ which originates from the merging of one ZS pair and one PS pair, and the infinite lifetime (namely infinite quality value in Figure S12, Supporting Information) arouses theoretically infinite SL—the sharp peak reaching $GD∼10^{6}$ in Figure 2c (Besides, the mathematical relationship *γ*_1*d*_↔*γ*_1_ between Equations (2) and (3) suggest similar degeneracy occurs for *κ* = 0 and *γ*_1*r*_ = 0, namely *γ*_1*d*_ = *γ*_1_ . And more details can be found in Figure S13, Supporting Information).

However, in such a two‐resonator system, the realization of singularities tends to require gain materials *γ*_2*d*_ < 0 or complex frequency *f*∈ℂ^[^ ^42^ ^]^ to compensate for loss, which encounters difficulties in on‐chip applications. When further increasing *γ*_2*d*_, the line‐width of transmission becomes finite (Figure S14, Supporting Information), altering BIC into electromagnetically induced transparency (EIT).^[^ ^38^, ^43^, ^44^, ^45^, ^46^, ^47^, ^48^ ^]^ In this process, the singular peak for SL reduces as $\max\left(GD\right)\propto \gamma_{2d}^{-1}$ (marked by blue circles in Figure 2c), but a window of the strong SL maintains. For FL, situation is similar as $-\min\left(GD\right)\propto \gamma_{2d}^{-1}$ (marked by red circles), but the convergence is more rapid owing to the high peaks contributed by the unmodulated TBS, corresponding to the constant $-\min\left(GD\right)\propto \gamma_{2d}^{0}$ (marked by red crosses).

**Figure 3:**
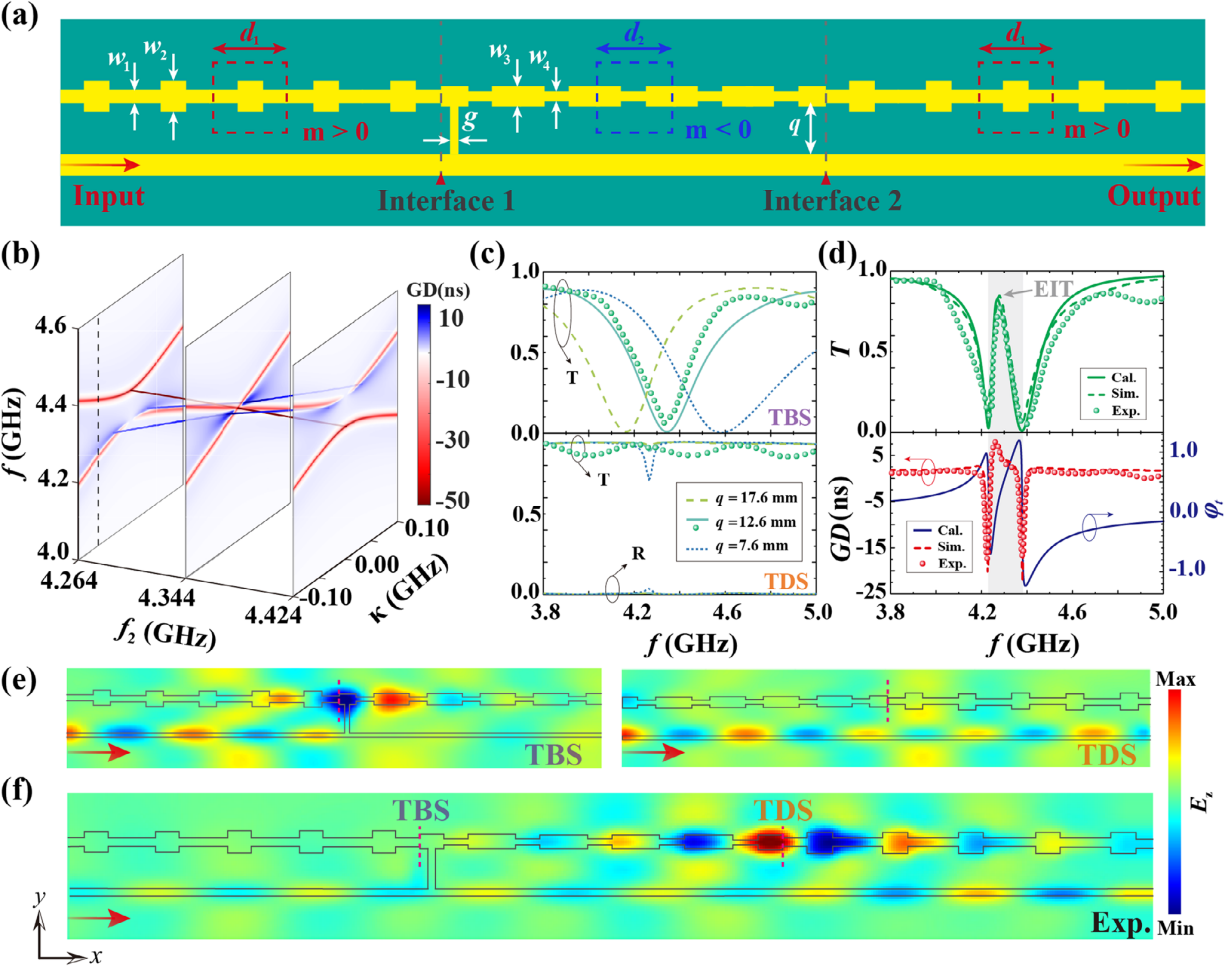
Experimental observation of the on‐chip topological EIT and switching between SL and FL. a) Schematic of the waveguide‐based on‐chip topological EIT sample containing a uniform waveguide (lower) and a composite waveguide with varying width (upper). Their unit cells are indicated by dashed boxes. b) *GD* slices for different *f*_2_ with *γ*_2*d*_ =  0.006 GHz. The other parameters are the same to Figure 2, and the experiment setup is marked by the dashed line. c) Simulated (lines) and measured (dots) spectra of the TBS and TDS. d) The top panel: the calculated (solid line), simulated (dashed line), and measured (dotted line) transmission spectra of the samples. The bottom panel: the calculated real parts of the susceptibility (solid line), simulated (dashed line), and measured (dotted line) *GD* spectra. e) Experimental measurement of *E_z_* distributions of on‐chip TBS (left) and TDS (right). f) Same as (e), but for an on‐chip topological molecule in the EIT window.

Based on the illustration of the analytical model, we developed a proof‐of‐concept based on the transmission lines, which exhibit high flexibility for constructing high‐performance metamaterials.^[^ ^49^, ^50^, ^51^, ^52^ ^]^ The effective waveguide‐based on‐chip topological structure in the transmission‐line platform to exhibit the topological EIT is shown in **Figure** **3**a. The inner waveguide with *m* < 0 was sandwiched between two topologically distinguished waveguides with *m* > 0, thereby forming two topological interfaces. The geometrical definitions of the associated width parameters were *w*_1_ =  3 mm, *w*_2_ =  7 mm, *w*_3_ =  4.8 mm, and *w*_4_ =  2 mm. The periods along the *x* direction were *d*_1_ = *d*_2_  =  *d*  =  24 mm. Using a branching waveguide with height *q* and width *g*  =  2 mm, the TBS at interface 1 could be directly excited by the waveguide below. The TDS at interface 2 should be excited by the near‐field coupling *κ* owing to the TBS. By increasing the number of unit cells in the middle waveguide with *m* < 0, *κ* decreased exponentially.

For such a lossy structure with *γ*_2*d*_ =  0.006 GHz, the minimum of *GD* (marked by the red line) is mainly dominated by the TBS—stable at *f*  = *f*_1_  and *κ*  =  0 when transforming *f*_2_ in Figure 3b. Intriguingly, the SL is enhanced through decreasing the detune |*f*_1_ − *f*_2_| to improve the spectral symmetry (Figure S14, Supporting Information). And the experiment setup is marked by the dashed line with *κ*  =   − 0.062 GHz henceforth. Furthermore, the computer simulation technology microwave studio software was used to obtain the spectra of the TBS and TDS as a function of *q* in Figure 3c, respectively. With the increase in *q*, the center frequency of TBS decreased. Crucially, the excitation pathway of the TDS is controlled by *q*. For a small *q* (e.g., 7.6 mm), the branching waveguide provides a direct excitation path to the upper interface, enabling direct excitation of the TDS (observed as a distinct resonance peak in transmission). However, for a sufficiently large *q* (e.g., 12.6 mm), this direct path is effectively eliminated. In this regime, the TDS at interface 2 cannot be directly excited by the waveguide below; its excitation occurs solely via near‐field coupling (*κ*) mediated by the TBS, matching well with the model proposed in Figure 1. Considering an appropriate height of branching waveguide *q* = 12.6 mm, the experimentally measured spectra of the TBS and TDS are represented by dotted lines in Figure 2b. The measured results from the vector network analyzer (VNA, Agilent N5222A) were consistent with the simulations. And the calculated EIT transmission and the susceptibility spectra are indicated by the solid lines in Figure 3d.

A transmission window marked by the gray arrow was observed in a broad opaque region with a peak transmittance of 80% at 4.28 GHz. Our data approximately reproduced the typical transmission spectra of the atomic EIT.^[^ ^53^ ^]^ Moreover, EIT shows remarkable potential for switching between SL and FL, because the susceptibility changes from anomalous dispersion to strong normal dispersion, resulting in a very slow group velocity.^[^ ^5^ ^]^ The simulated and measured transmission (*GD*) spectra are indicated by dashed lines and dots in the top (bottom) panel of Figure 3d, respectively (the time‐domain simulations in Figure S15, Supporting Information). To visualize the destructive interference between the two pathways, the electric field distributions were measured using near‐field scanning. The individual TBS (TDS) can (cannot) be directly excited by the waveguide below; thus, the energy will (will not) be localized at interface 1 (2), as shown in Figure 3e (the simulation results are shown in Figure S16, Supporting Information). However, the energy in the EIT window (4.25 GHz) are transferred from interface 1 (i.e., TBS) to interface 2 (i.e., TDS), and this process (demonstrated through changing the number of unit cells in the middle waveguide like Figure S17, Supporting Information) is like the topological state transfer in acoustic systems^[^ ^54^ ^]^ or phononic systems.^[^ ^55^ ^]^ (For better comparison, the corresponding simulated electric field distributions are shown in Figures S18, and S19, Supporting Information.

**Figure 4:**
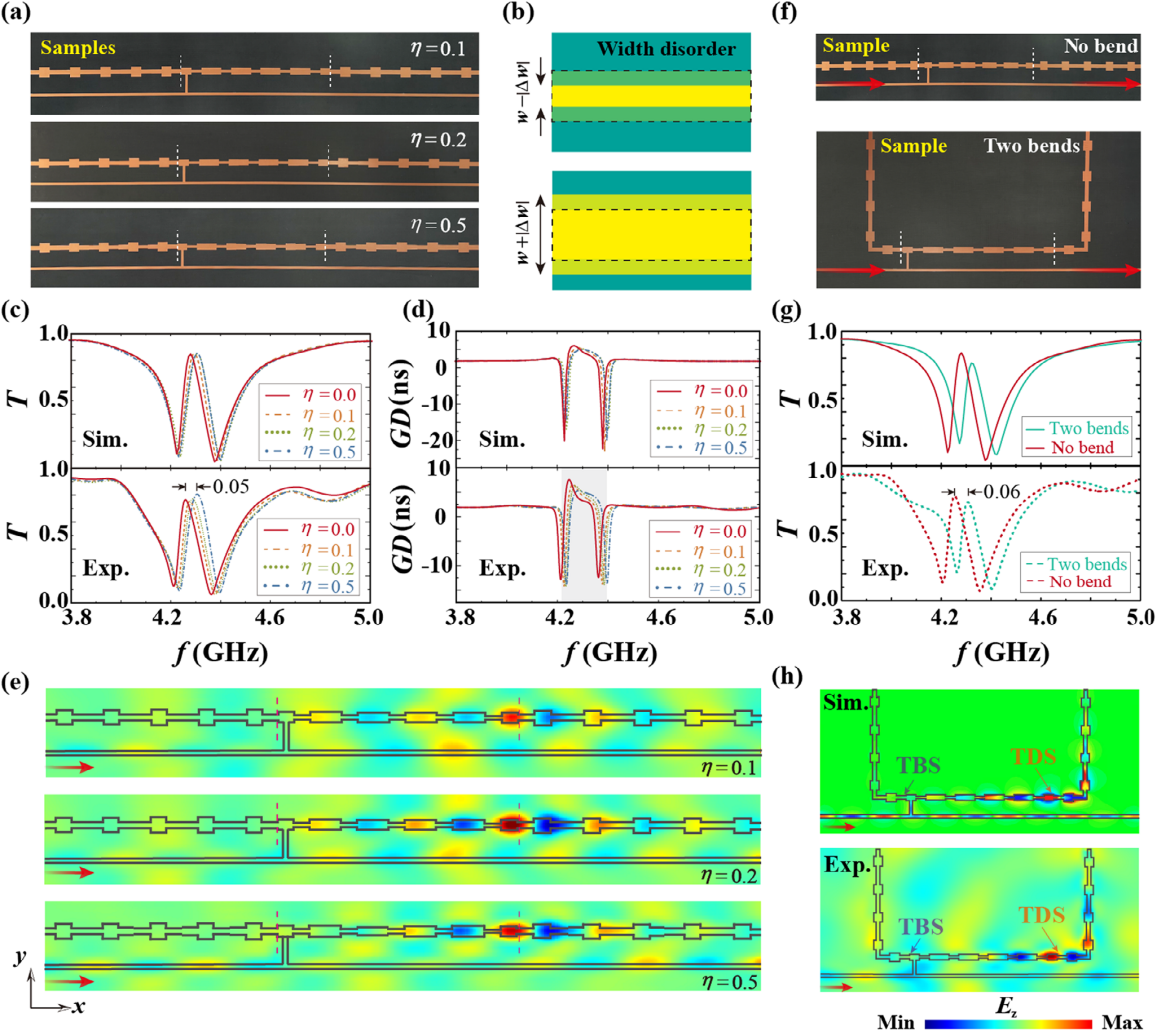
Experimental demonstration of the robustness of on‐chip topological EIT. a) Photographs of the fabricated samples with different disorder strengths: *η*  =  0.1 mm (top), 0.2 mm (middle), and 0.5 mm (bottom). b) Sketches of width disturbance with random increases and decreases in width are shown in the upper and lower panels, respectively. c) Simulated (top) and measured (bottom) transmission spectra for disordered samples. d) Similar to (c), but for the *GD* spectra. e) Experimental measurements of *E_z_* distributions of on‐chip topological molecules with different disorder strengths. f) Experimental photo of the waveguide‐based on‐chip topological EIT sample, no bend (top), and two 90° bends (bottom). g) Comparison of the simulated (top) and measured (bottom) transmission spectra of the sample with (green lines) and without (red lines) a bend. h) Simulated (top) and measured (bottom) electric field distributions of the on‐chip topological molecule with structure bending.

The emergence of a topologically protected EIT was validated experimentally. **Figure** **4**a shows the photographs of the three fabricated samples with different disorder strengths Δ*w*  =  *η* · *random*(− 1, 1), which was controlled by randomly increasing or decreasing the width of the discrete waveguide with the length of *d*/3  =  8 mm (Figure  4b). Compared with the simulated (top) and measured (bottom) transmission and *GD* spectra in Figure  4c,d, the EIT window was perfectly preserved under disorder strength *η*  =  0.1, 0.2, and 0.5 mm (Figure S20, Supporting Information). The EIT frequency only shifted by 0.05 GHz (i.e., 1.2% of the EIT window) even for the considered disorder strength *η*  =  0.5 mm (i.e., 40% of the waveguide width *w*_4_). To further understand the observed topological EIT phenomenon in the on‐chip system, the corresponding electric‐field distributions were measured, as shown in Figure  4e (the simulation results are shown in Figure S21, Supporting Information). Through comparisons of the field distributions of the EIT in the disordered topological structures, the destructive interference and localized field of the topological EIT were confirmed to be immune to width disorders.

Herein, the robustness of topological EIT for the structure bending is further verified. The photo of the waveguide‐based on‐chip topological EIT sample PC_B_‐PC_A_‐PC_B_ with two 90° bends is shown in Figure  4f (and several cases with different deformations in Figures S22, and S23, Supporting Information). The comparison of transmission spectra of the samples with and without bends is given in Figure  4g (the corresponding *GD* is shown in Figure S24, Supporting Information), where the simulated and measured transmission spectra are indicated by solid and dashed lines, respectively. From the transmission spectra, it can be found that the EIT window frequency of samples with structural bending has only a slight shift. Figure  4h displays the simulated and measured electric field distributions at the EIT window with 4.32 and 4.298 GHz, respectively. We can see that although there are two obvious structural bends, the energy transfer from the bright atom to the dark atom at the EIT window still remains (Figure S25, Supporting Information shows more details about the robustness of topological EIT to structural deformations).

## 3 Conclusion

In conclusion, we have introduced the concept of topological QI‐like effect in 1D waveguide‐based on‐chip samples. Taking topological EIT in the three‐level system as an example, the destructive interference between two excitation pathways is demonstrated experimentally through switching between SL and FL, which are robust against width disorders and bending deformations. In addition to its robustness against fabrication defects, the 1D on‐chip settings can offer significant advantages in miniaturization and integration. Compared with conventional 2D systems, they reserve plenty of space for design and modulation degree of freedom. As a result, more energy levels and excitation pathways are expected to join this platform to explore more complicated QI‐like effects with topological properties. This work can also be extended to other physical systems and has great potential to advance the field of QI‐like and topological physics and guide the design of novel on‐chip robust devices, such as optical switches, sensors, and optical storage.

## 4 Experimental Section

### Numerical Simulations

All full‐wave simulations finite‐element are performed using Computer Simulation Technology Microwave Studio software based on a finite integration method in the time domain. The structure is constructed on a commercially printed F4B circuit board, which has a relative permittivity and thickness of *ε*_*r*_ =  2.2 and *h*  =  1.6 mm, respectively. The loss tangent of the F4B substrate is 0.0079. The thickness and the conductivity of the metallic microstrip lines are set to be 18 µm and 5.8 × 10^7^ S m^−1^, respectively. The impedance of discrete port is 50 Ω, which is consistent with the experimental environment. Open boundary conditions are applied to all 3D directions.

### Experimental Implementation

In the experiment, the sample was placed on a 10‐cm‐thick foam substrate (permittivity *ε* ≈ 1) and mounted on an automatic translation stage with a scanning step of 1 mm to ensure precise field distribution measurements. A signal generated from port 1 of the vector network analyzer (Agilent PNA Network Analyzer N5222A) was transmitted to the input port of the sample, serving as the system's signal source. During the measurement, an electric probe (a small rod antenna, 5 mm in length) connected to port 2 of the VNA was positioned vertically 1 mm above the sample to measure the out‐of‐plane electric field component *E_z_*. To ensure measurement accuracy, full‐wave simulations were conducted to confirm that the measured field distribution closely aligned with the field distribution on the microstrip surface. The measured data were subsequently transmitted to the VNA. By analyzing the recorded field values, both the amplitude and phase distribution of the out‐of‐plane electric field *E_z_* were obtained, providing a foundation for further exploration of the topological EIT effect in the system.

## Conflict of Interest

The authors declare no conflict of interest.

## Supporting information



Supporting Information

## Data Availability

The data that support the findings of this study are available from the corresponding author upon reasonable request.
