# Supplementary material for: Topological Singularities and Edge‐State Coupling Enable Robust on‐Chip Slow Light
Source: Adv Sci (Weinh). 2025 Jul 2;12(36):e07226. doi: 10.1002/advs.202507226 (PMC12462937; doi:10.1002/advs.202507226)
Supplement: Supplementary file 1 — Supporting Information [file ADVS-12-e07226-s001.docx]

***Supporting Information***

**Topological Singularities and Edge-State Coupling Enable Robust On-Chip Slow Light**

Yuqian Wang ^1, †^, Shengyu Hu ^1†^, Zhiwei Guo ^1,^*****, Jie Jiang ^1^, Yaping Yang ^1^, Cuicui Lu ^2^, Hong Chen ^1^

^1^ MOE Key Laboratory of Advanced Micro-Structured Materials, School of Physics Science and Engineering, Tongji University, Shanghai 200092, China

^2^ Key Laboratory of Advanced Optoelectronic Quantum Architecture and Measurements of Ministry of Education, Beijing Key Laboratory of Nanophotonics and Ultrafine Optoelectronic Systems, School of Physics, Beijing Institute of Technology, Beijing 100081, China

* Corresponding author. Email: [2014guozhiwei@tongji.edu.cn](mailto:2014guozhiwei@tongji.edu.cn)

^†^ These authors contributed equally to this work.

**Outlines**

[S1. Topologically distinguished photonic crystal waveguides 1](#_Toc193392401)

[S2. Topological edge states with phase transition 7](#_Toc193392402)

[S3. Coupling between the topological edge states for two different configurations 8](#_Toc193392403)

[S4. PT-symmetry and broken PT-symmetry of the non-Hermitian systems compose of coupled edge states 11](#_Toc193392404)

[S5. Modulation of *GD* near singularities 17](#_Toc193392405)

[S6. *GD* obtained from the time-domain calculation 22](#_Toc193392406)

[S7. The EIT field distributions of the structure without disorders 22](#_Toc193392407)

[S8. Topological EIT realized by the inversed configuration 24](#_Toc193392408)

[S9. The topological EIT for different disorder configurations 26](#_Toc193392409)

[S10. The EIT field distributions of structures with different width disorder strength 28](#_Toc193392410)

[S11. Robustness of the topological EIT for the structural bending 29](#_Toc193392411)

[S12. Robustness of the topological EIT for the structural deformation 32](#_Toc193392412)

# S1. Topologically distinguished photonic crystal waveguides

For the waveguides formed by transmission lines, the effective refractive index can be flexibly controlled by the width of the waveguide. Considering a binary non-magnetic ($\mu_{A1}=\mu_{A2}=1$) photonic crystal (PC_A_): (A_1_A_2_A_1_)*_N_* realized by the waveguide structure, the with width of layers A_1_ and A_2_ is 4.8 mm and 2 mm, respectively. *N* denotes the number of the unit cells. Consequently, the corresponding effective permittivity of the layer layers A_1_ and A_2_ is approximately 2.4 and 1.2, respectively. The length of different layers is identical $d_{A1}=d_{A2}=8$ mm, and the total length of the symmetric unit cell is $\Lambda=d_{A2}$+${2d}_{A1}$=24 mm. The band structure of PC_A_ can be obtained as the dispersion relationship [S1]:

$\cos\left( q\Lambda\right)=\cos\left( k_{A1}2d_{A1} \right)\cos\left( k_{A2}d_{A2} \right)-\frac{1}{2}\left( \frac{z_{A1}}{z_{A2}}+\frac{z_{A2}}{z_{A1}} \right)\sin\left( k_{A1}2d_{A1} \right)\sin\left( k_{A2}d_{A2} \right)$, (S1.1)

where $q$ is the Bloch wavevector. $k_{A1}=\sqrt{\mu_{A1}\varepsilon_{A1}\omega/c}$ ($Z_{A1}=\sqrt{\mu_{A1}/\varepsilon_{A1}}$) and $k_{A2}=\sqrt{\mu_{A2}\varepsilon_{A2}\omega/c}$ ($Z_{A2}=\sqrt{\mu_{A2}/\varepsilon_{A2}}$) are the propagating wavevectors (impedance) in the layers A_1_ and A_2_, respectively. According to Equation (S1.1), the band structure of PC_A_ is shown in **Figure 1(a)**. The corresponding unit cell is shown by the inset. Especially, the first and second bandgaps are painted blue and pink, respectively.

It is well known that the topological invariant in one-dimensional systems can be determined by the quantized Zak phase of passbands ($\varsigma>0$), which is given by [S2]:

$\theta_{\varsigma}^{Zak}=\int_{-\pi/\Lambda}^{\pi/\Lambda} \left[ i\int_{unit cell} \varepsilon(x)u_{\varsigma,q}^{*}\partial_{q}u_{\varsigma,q}(x)dx \right]dq$, (S1.2)

where $\varepsilon(x)$ is the function of permittivity in space, and $u_{m,q}(x)$ represents the Bloch eigenfunction of electric field at the $\varsigma^{th}$ photonic passband with a Bloch wavevector $q$. Especially, the Zak phase of the lowest $0^{th}$ band ($\varsigma=0$) is determined by

$\exp\left( i\theta_{0}^{Zak} \right)=sgn \left[ 1-\varepsilon_{A1}\mu_{A2}/\varepsilon_{A2}\mu_{A1} \right]$, (S1.3)

According to Equations (S1.2) and (S1.3), the Zak phases of different isolated passbands are obtained, as labeled in the band structure in **Figure S1(a)**. It is noteworthy that the topological edge state in the bandgaps should be determined from the topological properties of bandgaps, which is obtained by the summation of the Zak phases of all the bands below this gap [S3, S4].

As an alternative, the topological properties of bandgaps can also be directly obtained from the effective mass [S5, S6]. Based on the approach of standard transfer-matrix, the characteristic matrix of the $j^{th}$ layer for the normalized incident light can be described as [S7, S8]:

$M_{j}=\left( \begin{matrix} cos\delta_{j} & iZ_{j}sin\delta_{j} \\ \frac{i}{Z_{j}}sin\delta_{j} & cos\delta_{j} \end{matrix} \right)$, (S1.4)

where $\delta_{j}=\sqrt{\varepsilon_{i}\mu_{i}}k_{0}d_{j}$ means the phase accumulation of propagation in the material. $k_{0}$ is the wave vector of light in the vacuum, respectively. The matrix $S=\left( \begin{matrix} S_{11} & S_{12} \\ S_{21} & S_{22} \end{matrix} \right)$ connecting the incident end and the exit end of the structure can be obtained from Equation S(1.4). The reflection coefficient of the structure can be written as $r=\frac{S_{11}-S_{12}+S_{21}-S_{22}}{S_{11}-S_{12}-S_{21}+S_{22}}$. Here, we discuss how to derive the effective parameters of bandgaps. For above mentioned PC_A_, the transfer matrix of the unit cell $M(A)=M_{A1}\cdot M_{A2}\cdot M_{A1}$ can be considered as the characteristic one of single-layered equivalent material

$M(A)=\left( \begin{matrix} M_{A11} & M_{A12} \\ M_{A21} & M_{A22} \end{matrix} \right)=\left( \begin{matrix} \cos\Gamma& iZ_{eff}\sin\Gamma\\ \frac{i}{Z_{eff}}\sin\Gamma& \cos\Gamma\end{matrix} \right)$, (S1.5)

where $\Gamma$and $Z_{eff}$ are the effective phase and the optical impedance of the equivalent material for the unit cell, respectively. Focusing on the bandgaps with strong reflection,$\left| M_{A11} \right|>1$ and $\Gamma=m\pi+i\xi$, where $\xi$ denotes the attenuation components in the structure. Furthermore, due to the real part doesn’t affect the topological properties of the band gap, Equation S(1.5) can be simplified as

$M(A)=\left( \begin{matrix} \cos i\xi& iZ_{eff}\sin i\xi\\ \frac{i}{Z_{eff}}\sin i\xi& \cos i\xi\end{matrix} \right)$, (S1.6)

The effective refractive index of the effective material is given by $n_{eff}=i\xi/k_{0}d$. According to the effective medium theory, the effective parameters are

$$\varepsilon_{eff}=\frac{n_{eff}}{Z_{eff}}$$

$\mu_{eff}=n_{eff}\cdot Z_{eff}$ (S1.7)

For a 1-D photonic system, the Maxwell equations can be written in the form of Dirac equation

$[-i\sigma_{x}\partial_{x}+m(x)\sigma_{z}+V(x)]\left( \begin{matrix} \sqrt{\varepsilon_{0}}E_{z} \\ \sqrt{\mu_{0}}H_{y} \end{matrix} \right)=E\left( \begin{matrix} \sqrt{\varepsilon_{0}}E_{z} \\ \sqrt{\mu_{0}}H_{y} \end{matrix} \right)$, (S1.8)

The effective mass $m(x)=(\omega/2c)[\varepsilon_{r}(x)-\mu_{r}(x)]$ of PC_A_ can be directly obtained from Equation (S1.5), as shown in **Figure S1(b)**. In addition, **Figures S1 (c, d)** give the corresponding transmission and the reflection phase spectra, respectively. Similarly, the properties of the topologically distinguished photonic crystal PC_B_: (B_1_B_2_B_1_)*_N_* in the transmission line platform are also studied. Considering the width of layers B_1_ and B_2_ is 3 mm and 7 mm, respectively. *N* denotes the number of the unit cells. Consequently, the corresponding effective permittivity of the layer layers B_1_ and B_2_ is approximately 1.5 and 2.9, respectively. The length of different layers is also the same as $d_{B1}=d_{B2}=8$ mm, and the total length of the symmetric unit cell is $\Lambda=d_{B2}+{2d}_{B1}$=24 mm. Same as **Figures S1(a-d)**, the band structure, effective electromagnetic parameters, reflection, and reflection phase are shown in **Figures S1(e-h)**, respectively. It can be clearly seen that the quantized invariant is topologically distinguished between PC_A_ and PC_B_.

**
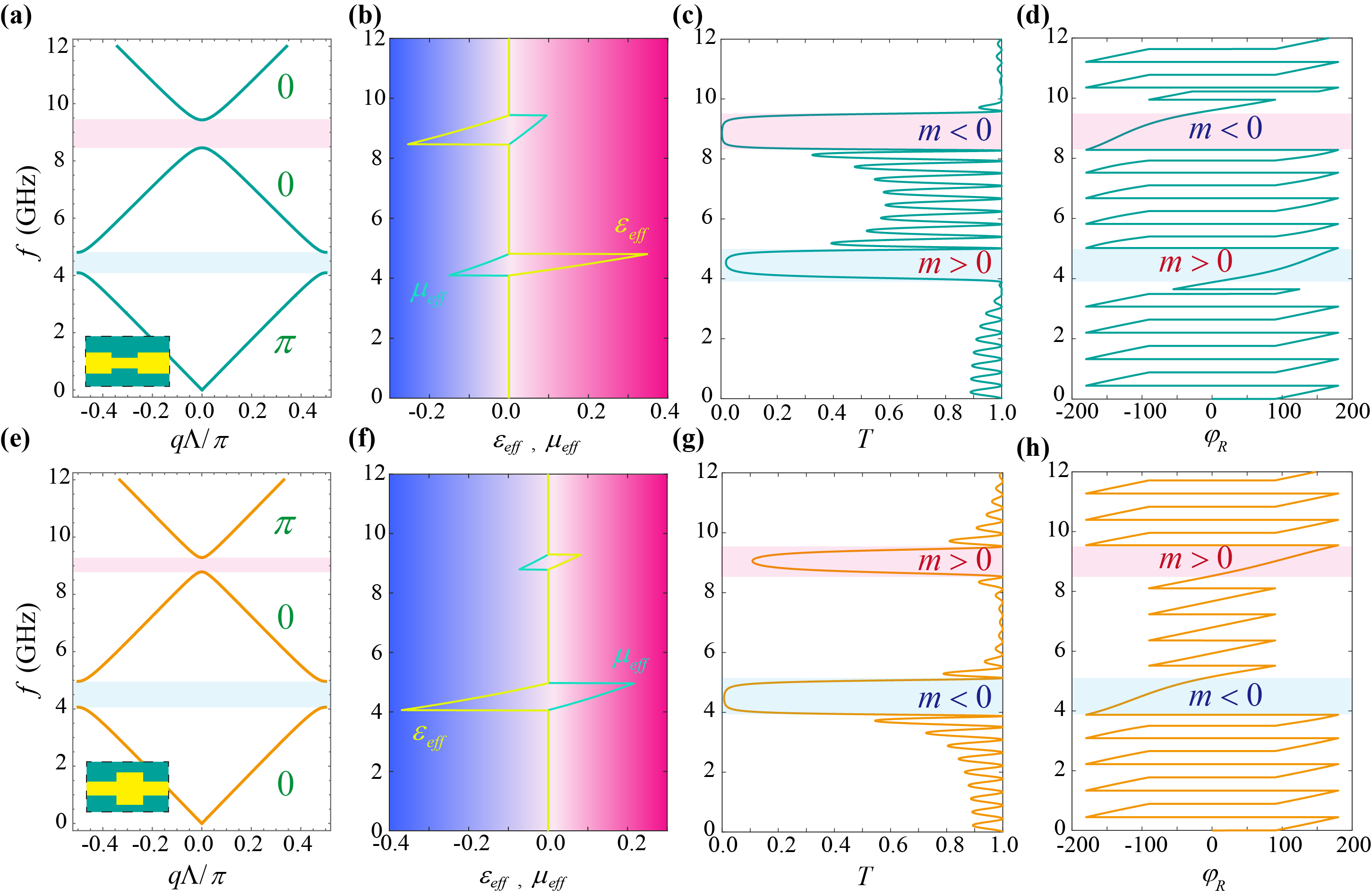
**

**Figure S1.** **Topological invariant of photonic crystals constructed by the on-chip transmission lines.** (a) Band structure of PC_A_, where the Zak phase is labeled near the corresponding passband. Inset shows the schematic of the unit cell of PC_A_. The first and second bandgaps are painted blue and pink. (b) Effective electromagnetic parameters of PC_A_, where the permittivity and permeability are marked by yellow and cyan. (c) Transmission spectra of PC_A_, and the effective mass of two bandgaps is marked in the bandgaps. (d) Reflection phase of PC_A._ (e)-(h) Similar to (a)-(d), but for the band structure, effective electromagnetic parameters, transmission spectra, and reflection phase spectra, respectively.

The topological properties of bandgaps of waveguide-based structures are verified from the full-wave simulations and measurements. For the finite PC_A_ with *N*=5, the schematic transmission line structure and the photo of the sample are shown in the top and bottom panels of **Figure S2(a)**. For comparison, the schematic transmission line structure and the photo of the sample for PC_B_ are also given in **Figure S2(b)**. Considering the electromagnetic waves are input from the left side of the structures, **Figures S2(c, d)** give the full-wave simulated and experimental measured reflection spectra, respectively. The first and second bandgaps can be clearly determined from the high-reflection regions, which are painted blue and pink, respectively. The band edges are also marked by the circles in **Figure S2(c)**. Overall, the measured results meet well the simulated ones, and the transportation properties are clearly observed.

**
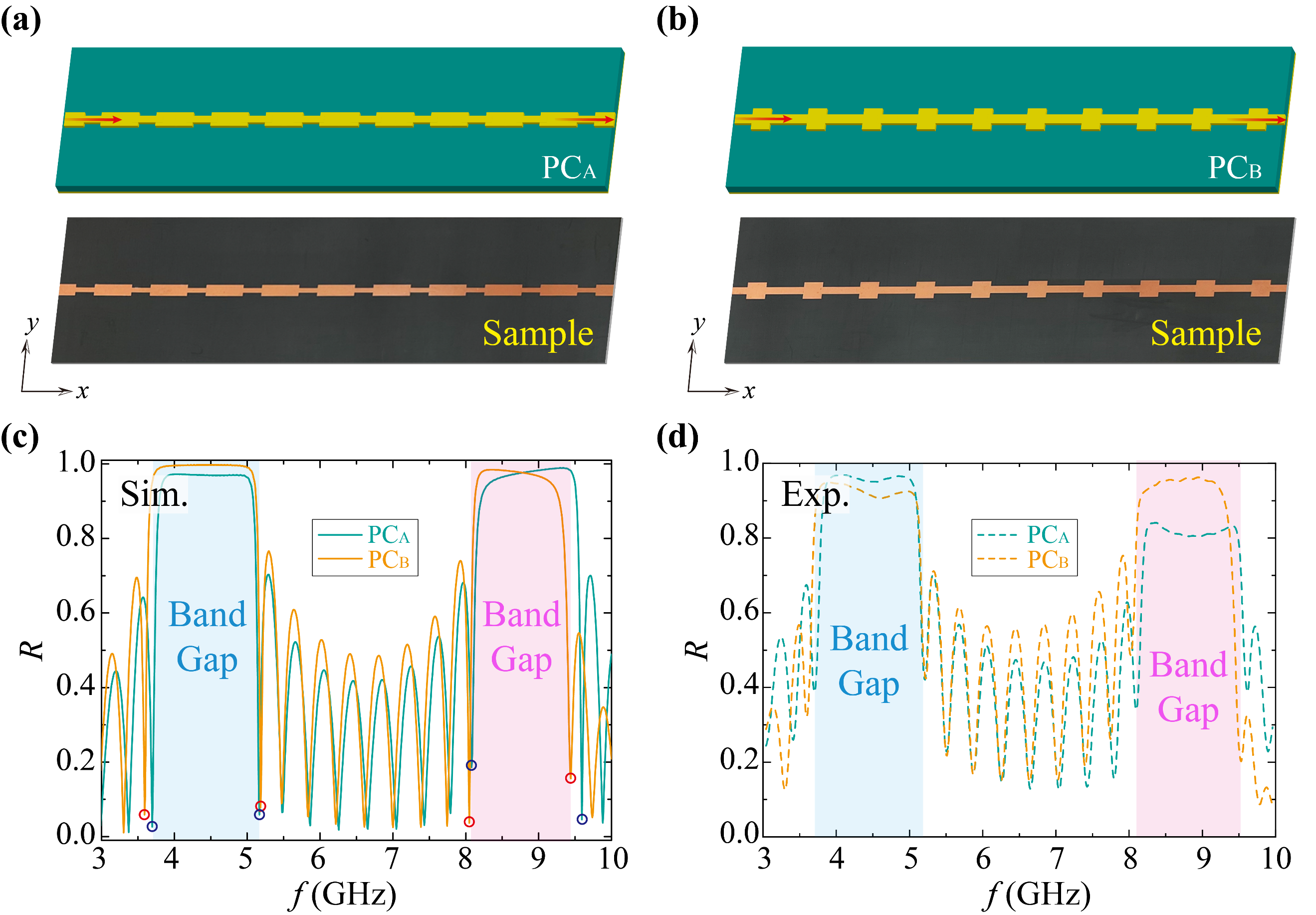
**

**Figure. S2.** **Reflection spectra of the topological structures in the transmission line platform.** (a) Schematic diagram (top) and sample photo (bottom) of the waveguide-based on-chip PC_A_. (b) Similar to (a), but for the waveguide-based on-chip PC_B_. Simulated (c) and measured (d) reflection spectra of the topological distinguished structures PC_A_ and PC_B_. The first and second bandgaps are painted blue and pink, respectively.

From **Figures S2(c, d)**, it can be found that the incident electromagnetic waves will be strongly reflected, and exponentially rapid decay in the structures. The corresponding simulated electric field distributions for PC_A_ and PC_B_ at 4.272 GHz are shown in **Figures S3 (a, b)**, respectively. The topological phase transition mentioned in **Figure S1** can also be determined from the band edge inversion. For the low-frequency band edges (LBEs) in the first bandgap of PC_A_, the simulated electric field distribution at 3.696 GHz (i.e., LBE1) and 5.168 GHz (i.e., LBE2) are shown in the top and bottom panels of **Figure S3(c)**, respectively. Similarly, the simulated electric field distribution of PC_B_ at 3.592 GHz (5.184 GHz) for the LBE1 (LBE2) is shown in the top (bottom) panel of **Figure S3(d)**. Compared **Figure S3(c)** with **Figure S3(d)**, it can be clearly seen that for the first bandgap of PC_A_, which corresponds to the effective mu-negative (MNG) bandgap with effective mass $m>0$, and the electric field of LBE1 (LBE2) is mainly localized at two ends (the center) of the structure. However, the first bandgap of PC_A_ corresponds to the effective epsilon-negative (ENG) bandgap with effective mass $m<0$, and the electric field of LBE2 (LBE1) is mainly localized at two ends (the center) of the structure. The same characteristic can also be applied to the other bandgaps. The simulated electric field distribution of PC_A_ and PC_B_ for the high-frequency band edges (HBEs) are shown in **Figures S3(e, f)**, respectively. Similar to the LBEs, the electric fields of HBE1 and HBE2 in effective MNG (ENG) bandgap are mainly localized at two ends and the center of the structure, respectively.

**
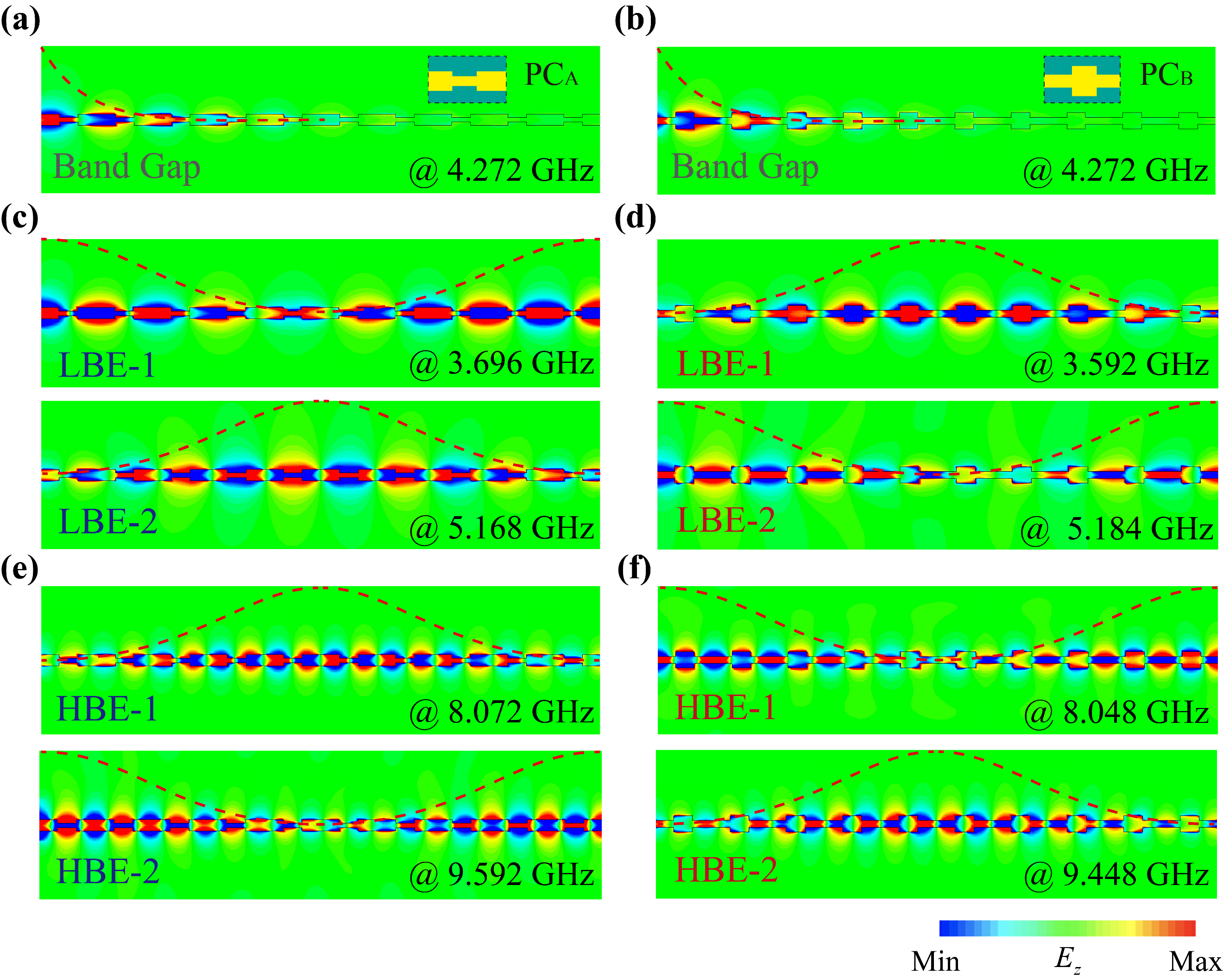
**

**Figure. S3.** **Comparison of electric field distributions of the topological distinguished structures.** The electric field distributions of PC_A_ (a) and PC_B_ (b) when the frequency falls in the first bandgap. The electric field distributions of PC_A_ (c) and PC_B_ (d) when the frequencies correspond to the low-frequency band edges (LEBs) marked in Figure S2(C). (e) (f) Same as (c) (d), but for the electric field distributions of PC_A_ (e) and PC_B_ at the high-frequency band edges (HBEs). The contour of the field distribution is represented by a red dashed curve.

# S2. Topological edge states with phase transition

From the above section, it can be found that there will be band inversion accompanied by changes in topological invariants from the designed PC_A_ to PC_B_. It is well known that if one combines these two PCs with different topological properties into a heterostructure, a topological edge state will form at the interface of the two PCs. The topological edge states used in the main text are uncovered in this section. The schematic diagram and the experimental photo of the waveguide-based topological heterostructure are shown in the top and bottom panels of **Figure S4(a)**. Especially, the interface between the left PC_A_ and right PC_B_ is marked by the white dashed line for see. Considering the electromagnetic waves incident from the left side of the structure, the full-wave simulated transmission spectra of individual PC_A_ (green solid line), individual PC_B_ (yellow solid line), and the combined heterostructure PC_A_-PC_B_ (pink solid line) are shown in **Figure S4(b)**. Furthermore, the corresponding electric field distributions of the topological edge states EG1 (4.264 GHz) and EG2 (8.904 GHz) in the first and second bandgaps are shown in **Figure S4(c)**. In contrast to the standing waves of the band edge shown in **Figure S3**, the topological edge states are strongly localized at the interface of the heterostructure, and exponentially decay into the structures on both sides. The contour of the field distribution is also represented by a red dashed curve. Same as the simulated results in **Figure 4(b)**, the experimentally measured transmission spectra of individual PC_A_ (green dashed line), individual PC_B_ (yellow dashed line), and the combined heterostructure PC_A_-PC_B_ (pink dashed line) are shown in **Figure 4(d)**. Take the measured low-frequency edge state EG1 at 4.267 GHz for example, the localized property is clearly demonstrated from the electric field distributions without and with phase are the top and bottom of **Figure S4(e)**.

**
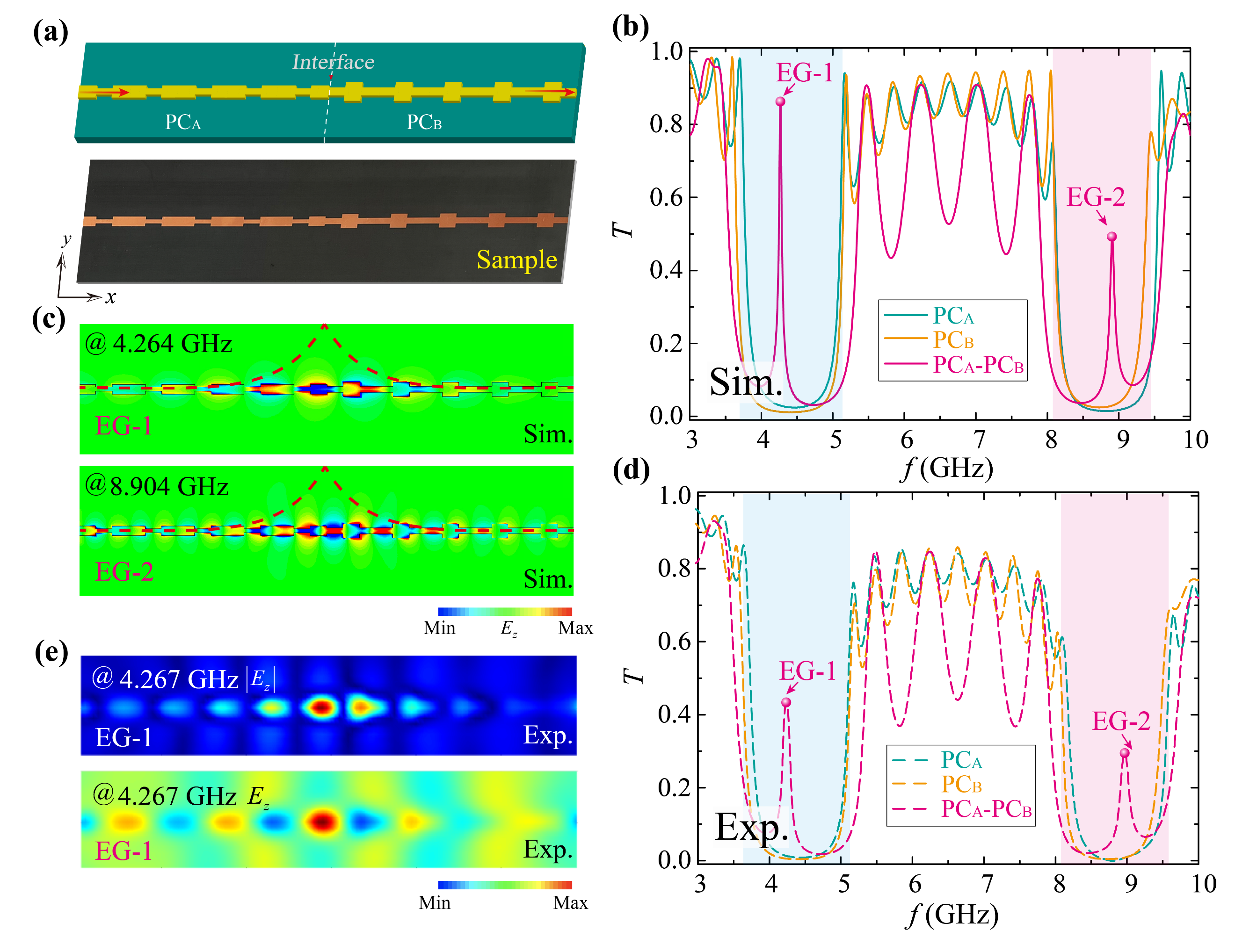
**

**Figure S4. Observation of the topological edge states in the waveguide-based topological heterostructure PC_A_-PC_B_.** (a) Schematic diagram (top) and experimental photo (bottom) of the waveguide-based topological heterostructure. (b) Simulated transmission spectra of the individual PC_A_, individual PC_B_, and the combined heterostructure PC_A_-PC_B_. Two edge states EG-1 and EG-2 in bandgaps are marked by the pink arrows. (c) Simulated electric field distributions of the edge states EG-1 (top) and EG-2 (bottom). (d) Same as (b), but for the measured transmission spectra. (e) Measured electric field distributions of the edge states EG-1 with (top) and without (bottom) phase.

# S3. Coupling between the topological edge states for two different configurations

For the on-chip waveguide-based topological edge states, the coupling strength and sign can be tuned by not only considering different bandgaps, but also changing the different configurations. Two configurations of near-field coupling heterostructures PC_A_-PC_B_-PC_A_ and PC_B_-PC_A_-PC_B_ are studied in this section, as schematically shown in **Figures S5(a, b)**, respectively. The simulated transmission spectra are shown in **Figure S5(c)**, where the asymmetric and symmetric splitting edge states (SEGs), namely SEG-1 (SEG-4) and SEG-2 (SEG-3) for the heterostructures PC_A_-PC_B_-PC_A_ (PC_B_-PC_A_-PC_B_) are marked by the red and blue spheres, respectively. The coupling properties of the SEGs are clearly given in the corresponding electric field distributions in **Figure S5(d)**. The asymmetric (symmetric) distribution in low-frequency mode while symmetric (asymmetric) distribution in high-frequency mode indicates the positive (negative) coupling for the heterostructures PC_A_-PC_B_-PC_A_ (PC_B_-PC_A_-PC_B_), respectively [S9–S11].

**
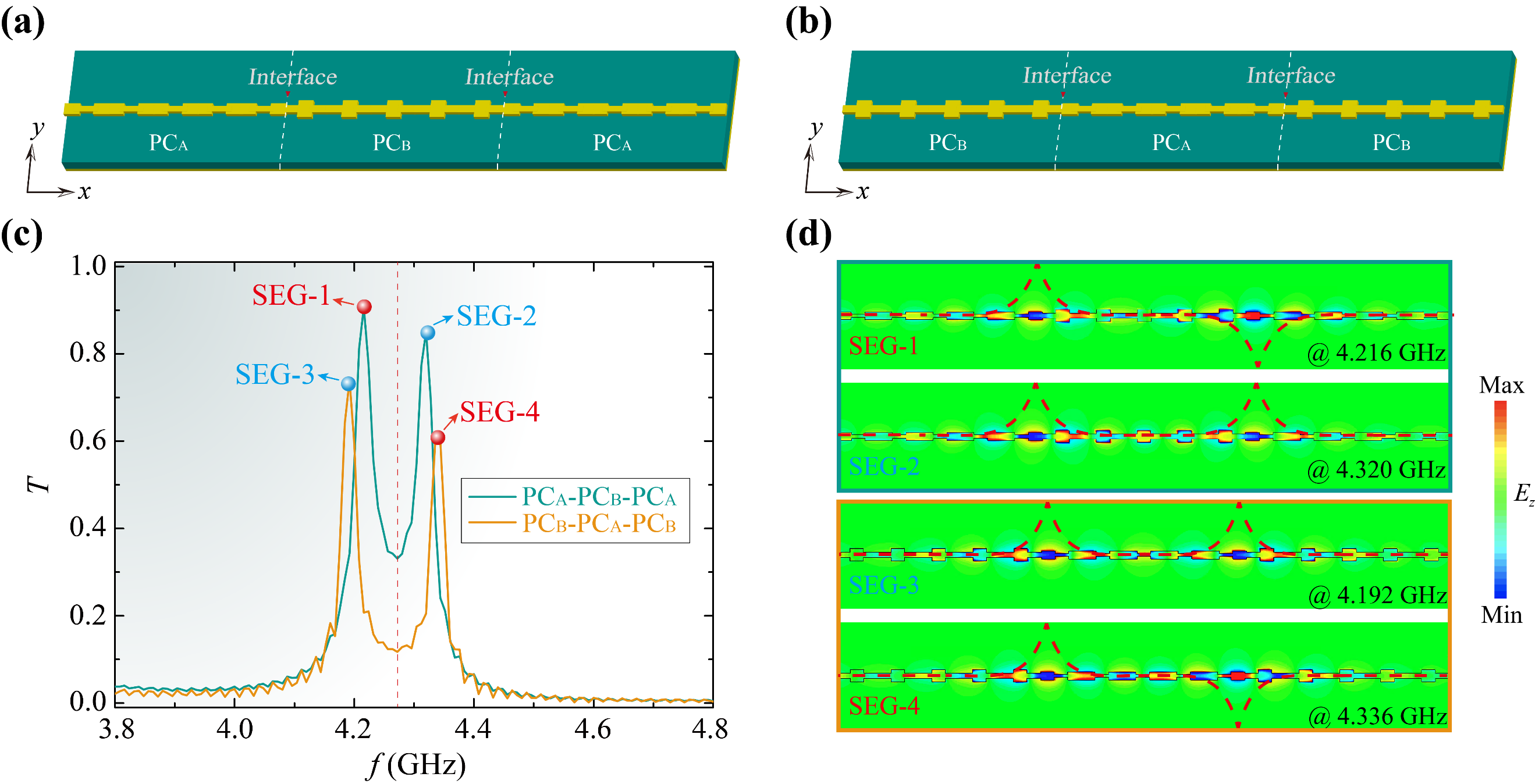
**

**Figure S5.** **Positive and negative near-field couplings between the edge states. Schematic diagram of the waveguide-based topological heterostructures**: (a) PC_A_-PC_B_-PC_A_ and (b) PC_B_-PC_A_-PC_B_. Two interfaces are marked by the dashed lines. (c) Simulated transmission spectra of the coupled edge states in PC_A_-PC_B_-PC_A_ (green line) and PC_B_-PC_A_-PC_B_ (yellow line). The asymmetric and symmetric splitting edge states (SEGs) are marked by red and blue spheres. (d) Simulated the electric field distributions of SEGs, where the asymmetric SEG-1 (SEG-4) and symmetric SEG-2 (SEG-3) in topological heterostructures PC_A_-PC_B_-PC_A_ (PC_B_-PC_A_-PC_B_) are shown in top (bottom) panels. The contour of the field distribution is represented by a red dashed curve.

Furthermore, the coupling properties of the edge states are demonstrated in **Figure S6**. The experimental samples correspond to the schematic diagram in **Figures S5(a, b)** are shown in **Figures S6(a, b)**, respectively. The measured transmission and transmission phase spectra are shown in **Figures S6(c, d)**, respectively. Similarly, the symmetric and asymmetric SEGs in two heterostructures are marked by the red and blue spheres. From the measured electric field distributions of SEGs in **Figures S6(e, f)**, the positive and negative couplings of edge states in the low-frequency bandgap are demonstrated for the heterostructures PC_A_-PC_B_-PC_A_ and PC_B_-PC_A_-PC_B_, respectively.

**
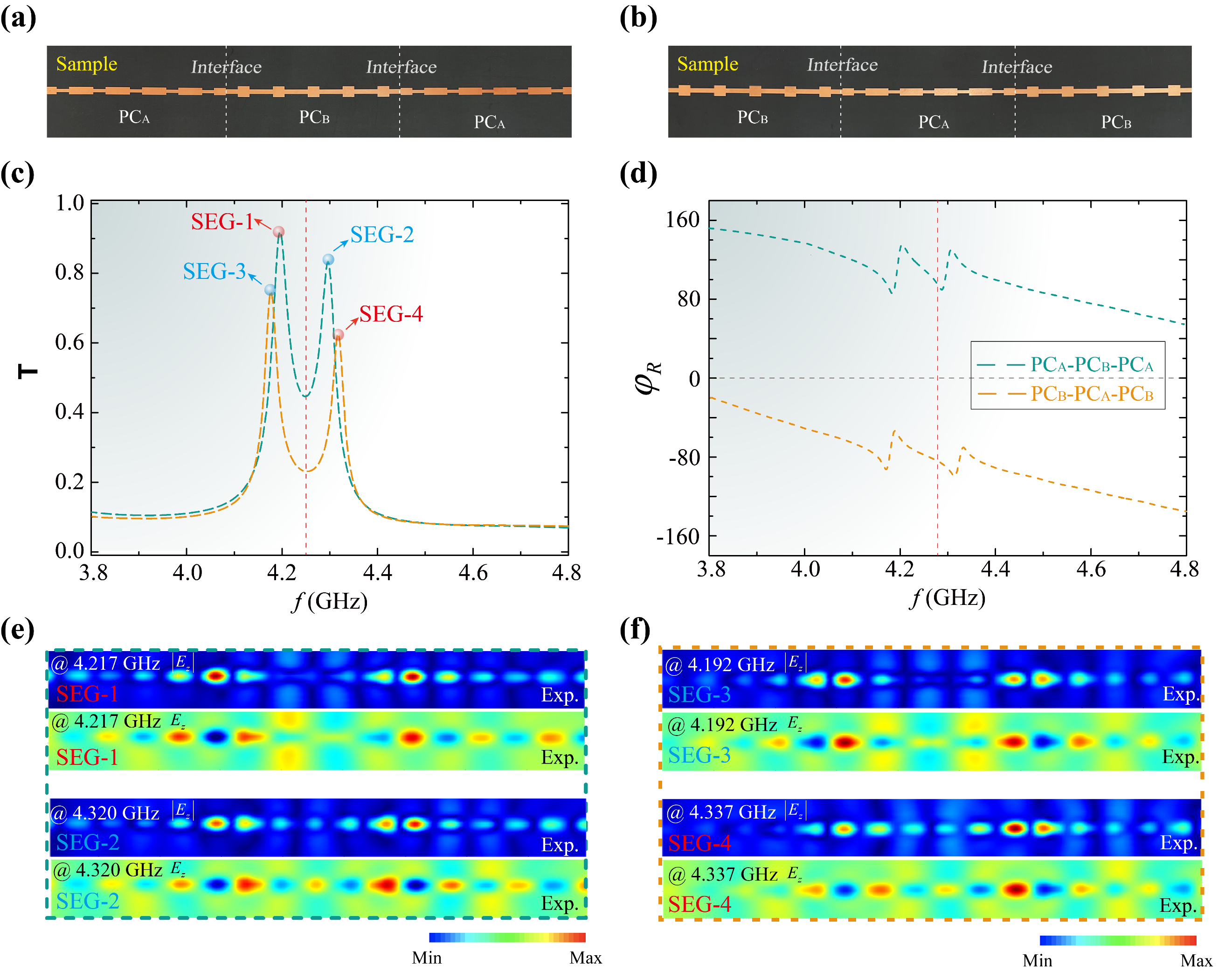
**

**Figure S6. Observation of the positive and negative near-field couplings between the edge states.** Experimental photos of the heterostructures PC_A_-PC_B_-PC_A_ (a) and PC_B_-PC_A_-PC_B_ (b). Two interfaces are marked by the white dashed lines. Measured transmission (c) and reflection phases (d) of the heterostructures PC_A_-PC_B_-PC_A_ and PC_B_-PC_A_-PC_B_. (e) Measured electric field distributions without (top) and with (bottom) phases of SEG-1 and SEG-2 in the low-frequency bandgap for the heterostructure PC_A_-PC_B_-PC_A_. (f) Similar to (E), but for the heterostructure PC_B_-PC_A_-PC_B_.

In addition to the different configurations, the coupling properties can also be controlled by changing the bandgaps. Considering edge sates in the high-frequency bandgap, the coupling properties in PC_A_-PC_B_-PC_A_ and PC_B_-PC_A_-PC_B_ are presented by the electric field distributions in **Figures S7(a, b)**, respectively. In contrast to the low-frequency bandgap, the coupling sign of edge states is reversed, that is SEG-1 (SEG-4) and SEG-2 (SEG-3) correspond to the symmetric and asymmetric distributions for the heterostructures PC_A_-PC_B_-PC_A_ (PC_B_-PC_A_-PC_B_), which are marked by the red and blue spheres, respectively.

**
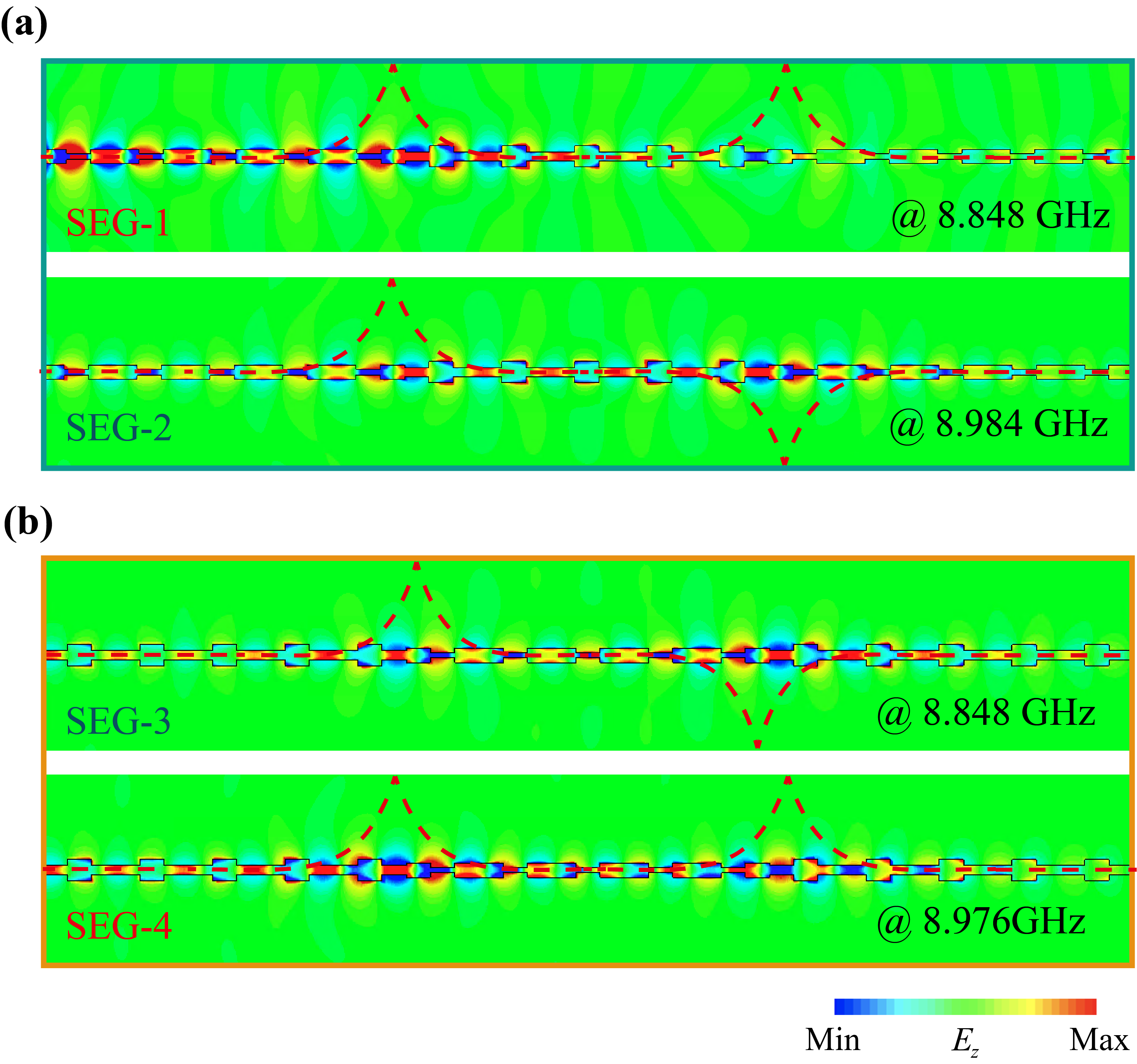
**

**Figure S7.** **Sign inversion of coupling coefficient in the high-frequency bandgaps.** Simulated the electric field distributions of SEGs, where the symmetric SEG-1 (SEG-4) and asymmetric SEG-2 (SEG-3) in topological heterostructures PC_A_-PC_B_-PC_A_ (PC_B_-PC_A_-PC_B_) are shown in top (a) (bottom (b)) panels. The contour of the field distribution is represented by a red dashed curve.

# S4. PT-symmetry and broken PT-symmetry of the non-Hermitian systems compose of coupled edge states

In contrast to the main text, the dynamic equation for the coupled edge states with direct excited can be modified as [S12]:

$$\frac{1}{2\pi}\frac{\partial a_{1}}{\partial t}=\left( if_{1}-\gamma_{1} \right)a_{1}+i\kappa a_{2}+i\sqrt{2\gamma_{i}}S_{in}$$

$\frac{1}{2\pi}\frac{\partial a_{2}}{\partial t}=\left( if_{2}-\gamma_{2} \right)a_{2}+i\kappa a_{1}$ (S4. 1)

Considering the excitation-free condition ($S_{in}=0$), for the edge states, the eigenvalue equation is written as:

$\boldsymbol{H}\left( \begin{matrix} a_{1} \\ a_{2} \end{matrix} \right)=\omega\left( \begin{matrix} a_{1} \\ a_{2} \end{matrix} \right)$, (S4. 2)

where the effective Hamiltonian corresponds to:

$\boldsymbol{H}=\left( \begin{matrix} f_{1}-i\gamma_{1} & \kappa\\ \kappa& f_{2}-i\gamma_{2} \end{matrix} \right)$. (S4. 3)

For $\gamma_{1}=\gamma_{2}$, $\boldsymbol{H}=\left[ \frac{f_{1}+f_{2}}{2}-i\gamma_{1} \right]\sigma_{0}+\kappa\sigma_{x}+\frac{\delta}{2}\sigma_{z}$ with the Pauli matrix $\sigma_{i}$ ($i=0,x,y,z$) and the frequency detuning $\delta=f_{1}-f_{2}$ corresponding to a Dirac point (DP) when $\kappa=0$ and $\delta=0$. Take the space $\kappa-\delta$ in **Figure S8(a)** as an example. Since $\kappa=0$ corresponds to the decoupled TBS and TDS, the real part of two eigenfrequencies of the Hamiltonian are $f_{1}$ and $f_{2}$, coincident with TBS and TDS. Here we consider $f_{1}=4.344$ GHz is fixed, and the Dirac cone has a flat band along the direction of $\delta$, corresponding to the critical type III DP [S13]. Besides, the imaginary parts are $\gamma_{1}$ independent of $\kappa$ and $\delta$, as is shown in **Figure S8(b)**. Furthermore, the original DP is spilt into two EPs appearing on the Riemann surface.

Considering the zero-flection condition ($S_{-}=-S_{in}+\sqrt{2\gamma_{1}}a_{1}$) for the edge states, the eigenvalue equation from **Equation (S4.1)** is written as:

$\boldsymbol{H}\left( \begin{matrix} a_{1} \\ a_{2} \end{matrix} \right)=\omega\left( \begin{matrix} a_{1} \\ a_{2} \end{matrix} \right)$, (S4. 4)

where the effective Hamiltonian corresponds to [S14, S15]:

$\boldsymbol{H}=\left( \begin{matrix} f_{1}-i\gamma_{1} & \kappa\\ \kappa& f_{2}+i\gamma_{2} \end{matrix} \right)$. (S4. 5)

**
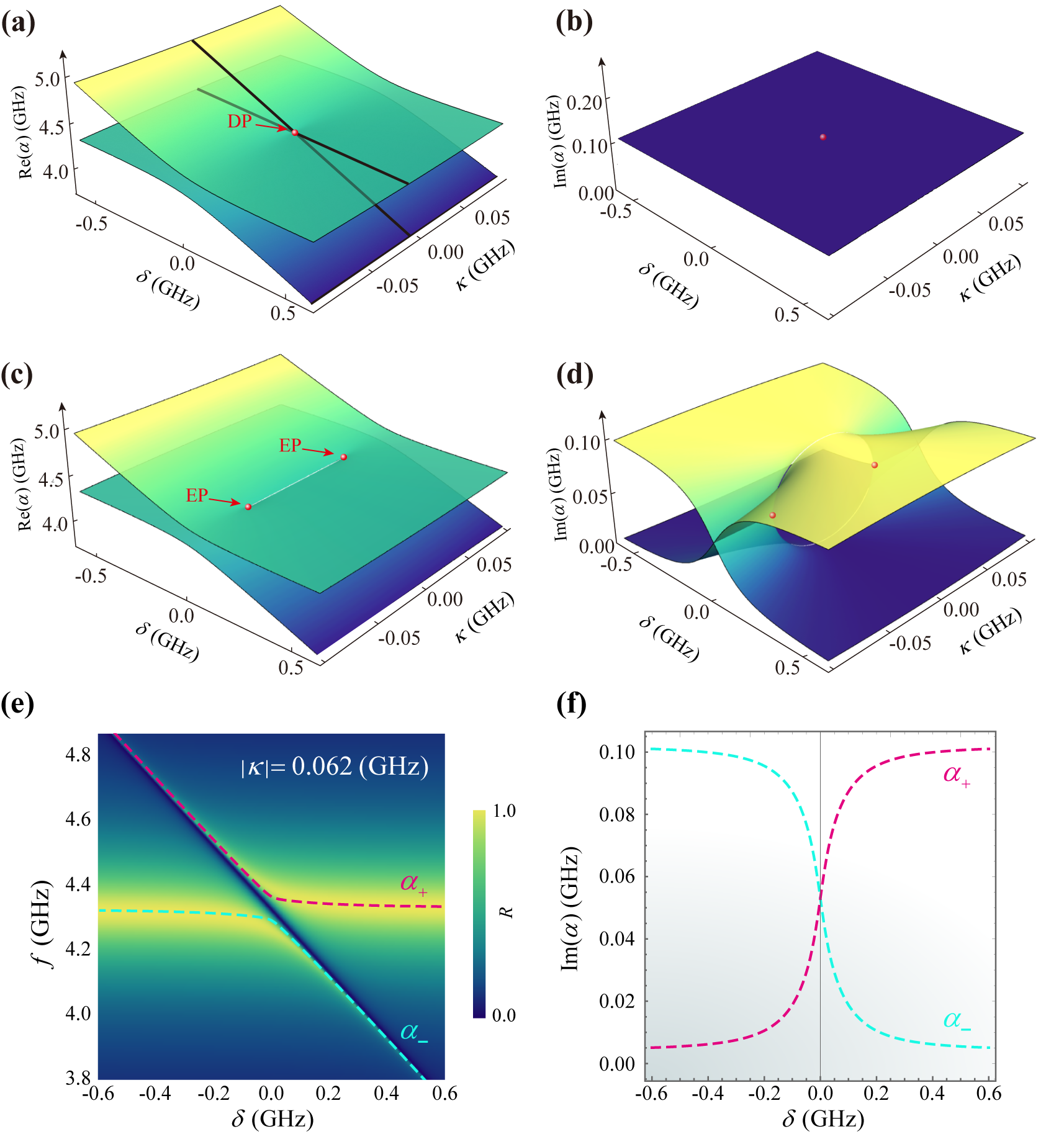
**

**Figure S8.** **Fundamental characteristics of the topological EIT structure.** Evolution of the real parts (a) and imaginary parts (b) of the eigenfrequencies as a function of frequency detuning ($\delta$) and coherent coupling ($\kappa$). The DP is marked by the red dot. For clarity, the eigenvalues at $\kappa=0$ are highlighted by black lines in (a), which possess linear dispersion along the dimension of δ and correspond to the critical type III DP. Evolution of the real parts (c) and imaginary parts (d) of the eigenfrequencies as a function of frequency detuning ($\delta$) and coherent coupling ($\kappa$) in the $\Lambda$-type three-level system. Two exceptional points (EPs) are marked by the red dots. (e) Reflection spectra as a function of $\delta$. The pink (cyan) dashed line corresponds to the real part of the high- (low-) eigenfrequency when the coupling strength is fixed at $\left| \kappa\right|\boldsymbol{=}$ 0.062 GHz. (f) Corresponding imaginary parts of the splitting eigenfrequencies.

Furthermore, the original DP is spilt into two exceptional points (EPs) appearing on the Riemann surface under the conditions of $\kappa=\pm\left( \gamma_{1}+\gamma_{2}+i\delta\right)/2$, where the eigenvalues and corresponding eigenvectors simultaneously coalesce. Here, considering the actual parameter $f_{1}\boldsymbol{=}$4.344 GHz, the evolution of the real and imaginary parts of the eigenfrequencies as a function of the frequency detuning $\delta$ and coherent coupling $\kappa$ are shown in **Figures S8(c, d)**, respectively. In particular, the EPs for the case of $\delta=0$ separated the regions of the real and imaginary parts, marked by red dots. When the frequency detuning cannot be ignored ($\delta\neq0$), the imaginary parts of the eigenfrequencies always split, indicating that the splitting modes after the level repulsion of the coherent coupling mechanism yield different quality (*Q*-) factors. Based on Equation (1), the reflection coefficient of the three-level system was obtained using $R=i\sqrt{\gamma_{1}}a_{1}/S_{in}$. For a fixed near-field coupling strength $\kappa=$ 0.062 GHz, the reflection coefficient as a function of $\delta$ and the frequency $f$ is shown in **Figure S8(e)**. The calculated eigenfrequency dispersions are plotted as dashed curves. It is well known that the lower the imaginary part of the eigenfrequency, the higher its corresponding *Q*-factor. The different *Q*-factor (or linewidths) of the two splitting modes in **Figure S8(e)** can be explained based on the imaginary parts of eigenfrequencies shown in **Figure S8(f)**.

For the direct excitation, the resonance frequency between two edge states localized at interfaces 1 and 2 is the same ($f_{1}=f_{2}$), and the radiative loss is also the same ($\gamma_{1}=\gamma_{2}$). Consequently, the PT-symmetry of the second-order non-Hermitian system is verified for the effective Hamiltonian i.e., $(PT)\boldsymbol{H}(PT)^{-1}\boldsymbol{=}P\boldsymbol{H}^{\boldsymbol{*}}P\boldsymbol{=H}$ [S14].

Especially, for the configuration of PC_A_-PC_B_-PC_A_, the PT-symmetric transition of the second-order non-Hermitian system realized by the coupled edge states are studied by changing the number of the unit cell *N* in the interlayer PC_B_. The simulated transmission spectra of the non-Hermitian systems as a function of *N* are shown in **Figure S9(a)**. It can be clearly seen that with the increase of *N*, the splitting edge modes gradually approach and coalesce at the exceptional point (EP). In particular, EPs separate the PT-symmetry and broken PT-symmetry in the phase diagram. **Figure S9(b)** gives the simulated coupling strength of the edge states depends on *N*. With the increase of *N*, the coupling strength will decay exponentially. Based on the extracted coupling parameters, the calculated evolution of real parts of eigenfrequencies in the effective second-order PT-symmetric non-Hermitian system as a function of the *N* is shown by the solid line in **Figure S9(c)**. The full-wave simulated eigenfrequencies meet well with calculated ones, as shown by the pink spheres in **Figure S9(c)**.

**
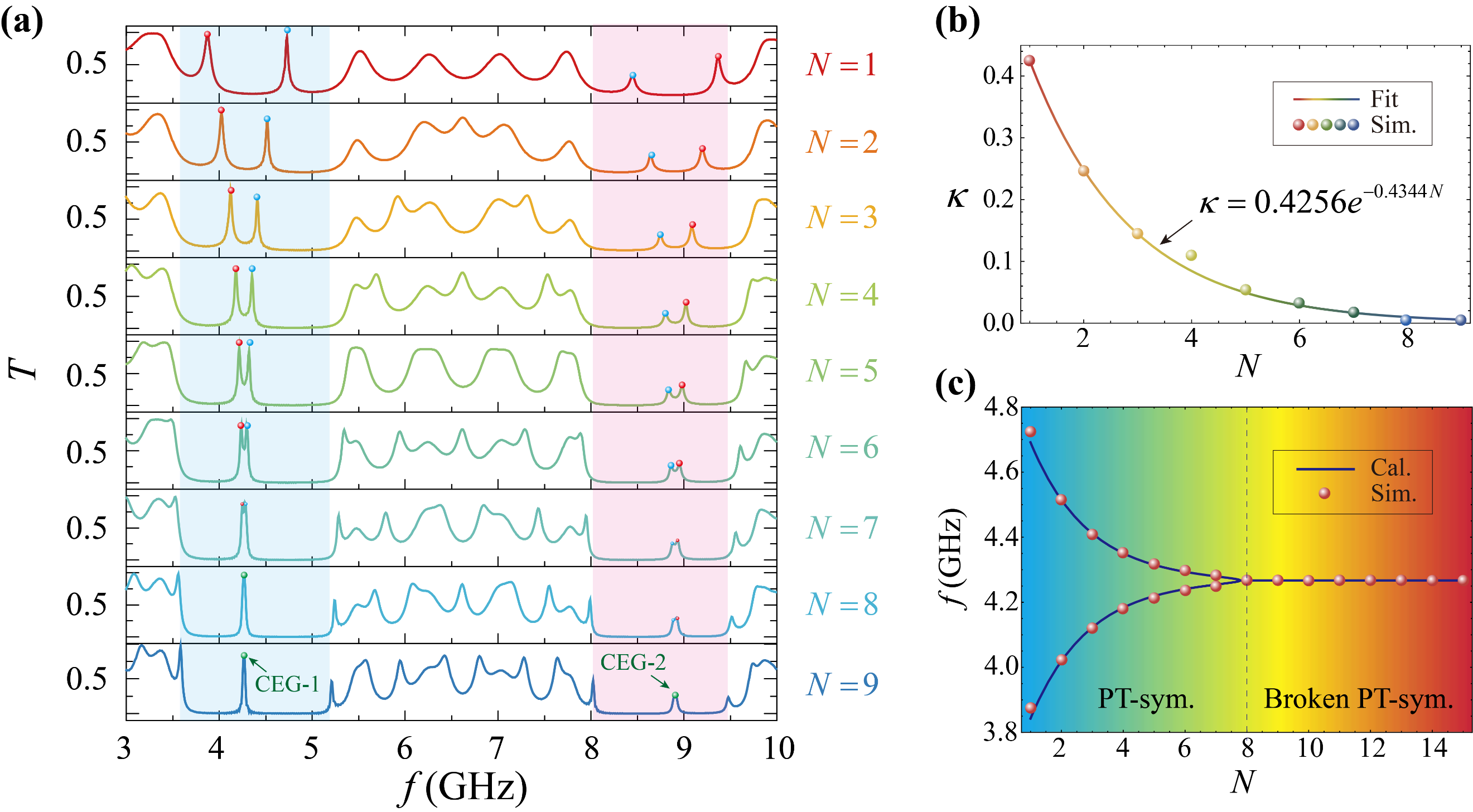
**

**Figure S9.** **PT-symmetry transition of the non-Hermitian system composed of coupled edge states.** (a) Simulated transmission spectra of the heterostructure PC_A_-PC_B_-PC_A_ as a function of the number of the unit cell *N* in the interlayer PC_B_. The symmetric and asymmetric edge states are marked by the red and blue spheres, respectively. The coupled edge states (CEGs) for *N*=9 in two bandgaps are marked by CEG-1 and CEG-2, respectively. (b) The near-field coupling coefficient as a function of *N*. (c) Evolution of the calculated (simulated) real parts of eigenfrequencies in the effective second-order PT-symmetric non-Hermitian system as a function of the *N*, which is marked by the blue solid line (pink spheres).

The transmission properties of the effective non-Hermitian system with PT-symmetry have been systematically studied with *N*=5 in **Figures S5** and **S6**. The coupling properties of edge states in the effective non-Hermitian system with broken PT-symmetry are shown in **Figure S10**. For the large *N*=9, the coupling strength between two edge states is weak, which corresponds to the broken PT-symmetric phase. The schematic diagram and experimental photo of the waveguide-based topological heterostructure with *N*=9 in interlayer PCB are shown in the top and bottom panels of **Figure S10(a)**, respectively. From the simulated transmission spectra in **Figure S9(a)**, the corresponding electric field distributions of CLE-1 and CLE-2 are shown in the top and bottom panels of **Figure S10(b)**, respectively. Moreover, the measured electric field distributions and transmission spectra in **Figures S10(c-e)** are meet well the simulated ones.

**
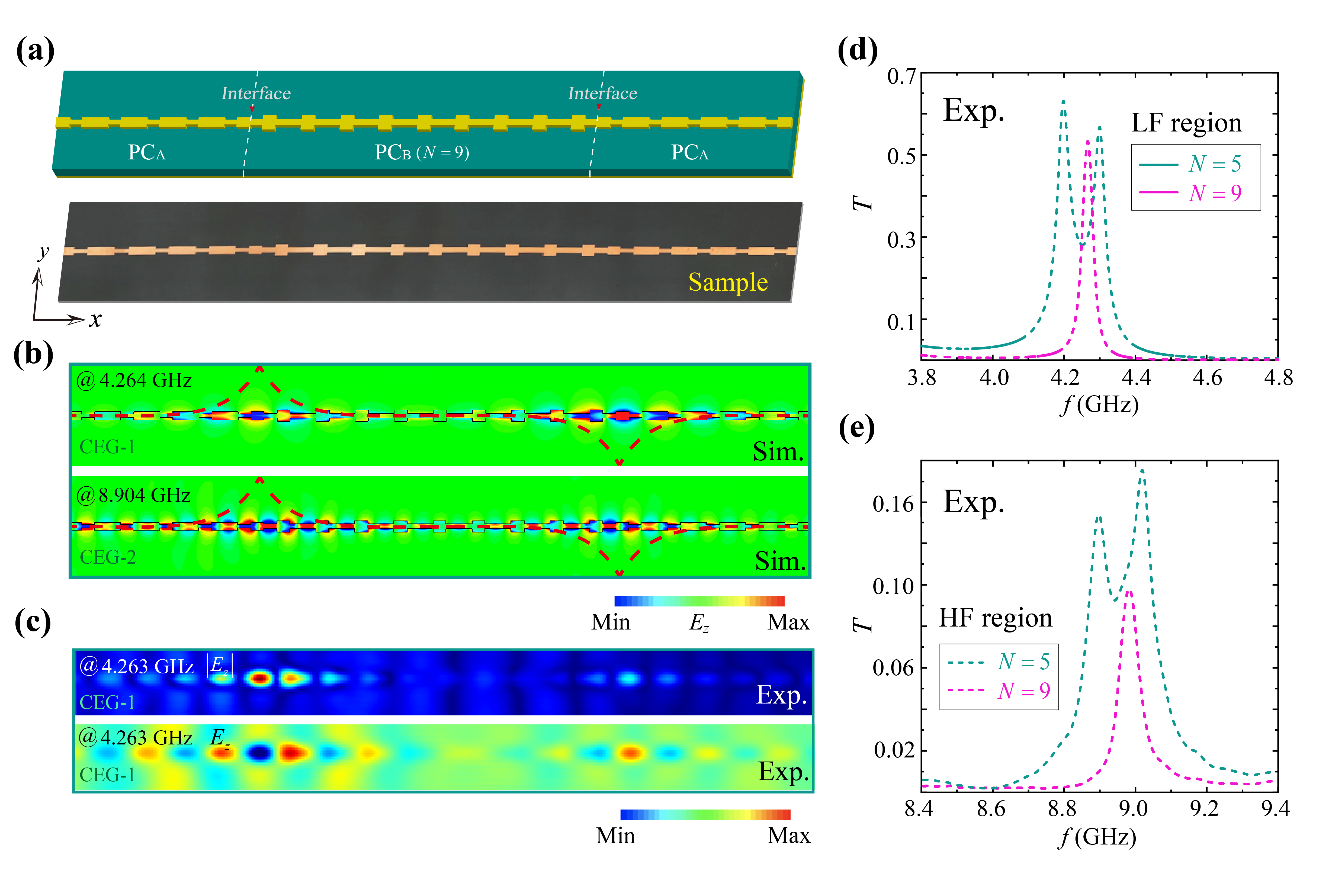
**

**Figure S10.** **Observation of broken PT-symmetry of the non-Hermitian systems composed of coupled edge states.** (a) Schematic diagram (top) and experimental photo (bottom) of the waveguide-based topological heterostructure PC_A_-PC_B_-PC_A_ with *N*=9 in interlayer PC_B_. (b) Simulated electric field distributions of the coupled edge states (CEGs): (top) CEG-1 in the low-frequency bandgap, and (bottom) CEG-2 in the high-frequency bandgap. (c) Measured electric field distributions of the CEG-1 without and with phase in the top and bottom panels, respectively. (d) Comparison of the measured low-frequency transmission spectra of the heterostructure PC_A_-PC_B_-PC_A_ with *N*=5 (green line) and *N*=9 (pink line). (e) Similar to (D), but for the high-frequency transmission spectra.

# S5. Modulation of *GD* near singularities

**Figure S11(a)** provides the diagram of $\gamma_{2d}=-0.001$ GHz, which is an important slice in the evolution of singularities in **Figure 2(a)**. Coming from the bound state in the continuum (BIC) for $\gamma_{2d}=0$ GHz, the pole singularity (PS) pair move faster than the zero singularity (ZS) pair. Noteworthy, there is a susceptibility jump line connecting PS and ZS, which can be described by the condition $(f-f_{1})(f-f_{2})^{2}+\gamma_{2d}\gamma_{2d}(f-f_{1})-\kappa^{2}(f-f_{2})=0$. Focus on the susceptibility along the dashed line in **Figure S11(a)**, a $2\pi$ jump appears near $f=4.24$ GHz in **Figure S11(b)**, originating from the constraint $\varphi_{t}\in[0,2\pi]$.

*
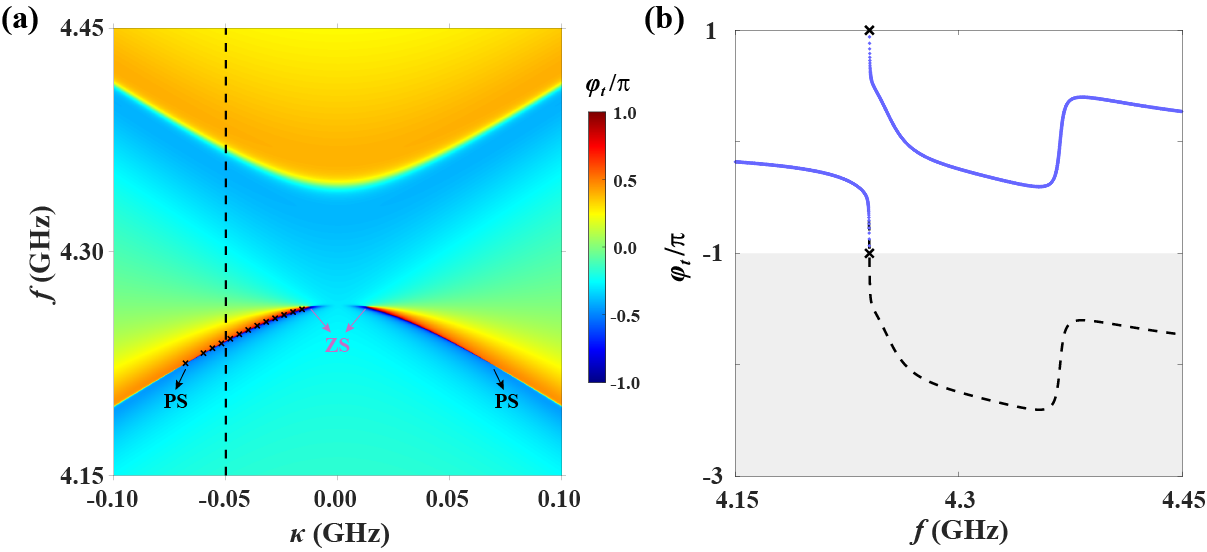
*

**Figure S11. Susceptibility jump between PS and ZS.** (a) Susceptibility diagram with $\gamma_{2d}=-0.001$ GHz. The other parameters are same to Figure 2. The crosses mark the susceptibility jump line connecting PS and ZS. For simplification, only the part of $\kappa<0$ is plotted. (b) Susceptibility spectrum along the dashed line in (A). The blue solid (black dashed) line corresponds to the spectrum before (after) translation.

**
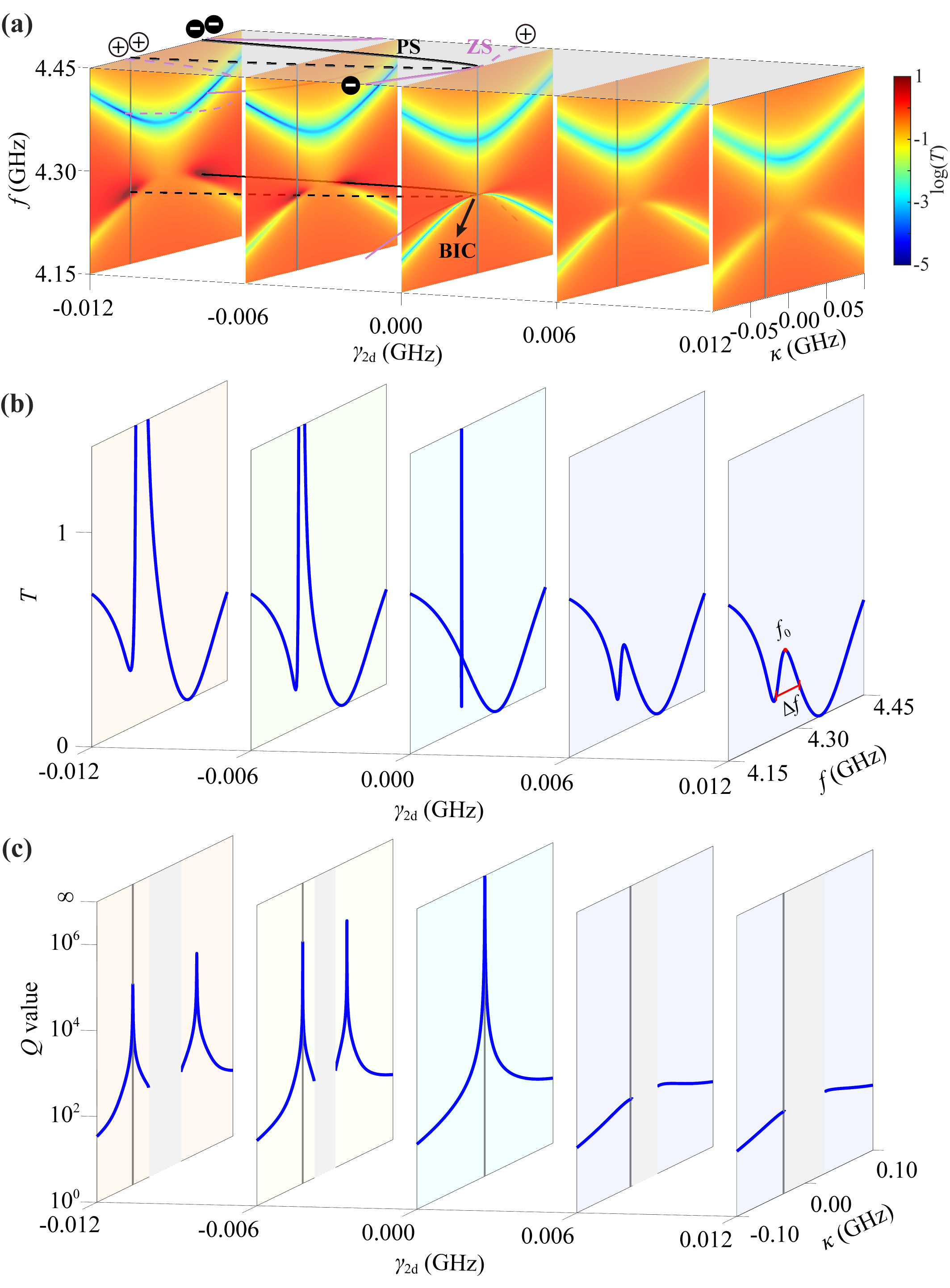
**

**Figure S12.** **Quality value near topological singularities.** (a) Transmission slices with different $\gamma_{2d}$, and the trajectories of singularities are projected to the top plane. (b) On the slices, the discrete spectra of transmission related to the solid gray lines in (a). Through the center frequency $f_{0}$ and the full width $\Delta f$ at half maximum, the quality value $Q=f_{0}/\Delta f$ can be obtained. (c) Related Q value slices, where the shaded regions correspond to $\Delta f$ is interfered with the neighbor transmission dips. Here $f_{1}=4.344$ GHz, $f_{2}=4.264$ GHz, $\gamma_{1d}=0.003$ GHz, and $\gamma_{1r}=0.11$ GHz are fixed.

Based on the transmission spectra in **Figure S12(a)**, we focus on the evolution along the dimension of frequency like the solid gray lines. Through extracting the center frequency $f_{0}$ and the full width $\Delta f$ at half maximum in each spectrum [like **Figure S12(b)**], the quality value $Q=f_{0}/\Delta f$ can be obtained. As is shown in slices of **Figure S12(c)**, two peaks of quality values appear at the pair of pole singularities [PSs, corresponding to the black trajectories on the top plane of **Figure S12(a)**] when $\gamma_{2d}<0$. At $\gamma_{2d}=0$, a bound state in the continuum (BIC) originates from merging of one ZS pair and one PS pair, arousing a diverging quality value, which corresponds to an infinite lifetime. When further increasing $\gamma_{2d}$, the quality value becomes finite (about ${10}^{2}$), altering BIC into EIT.


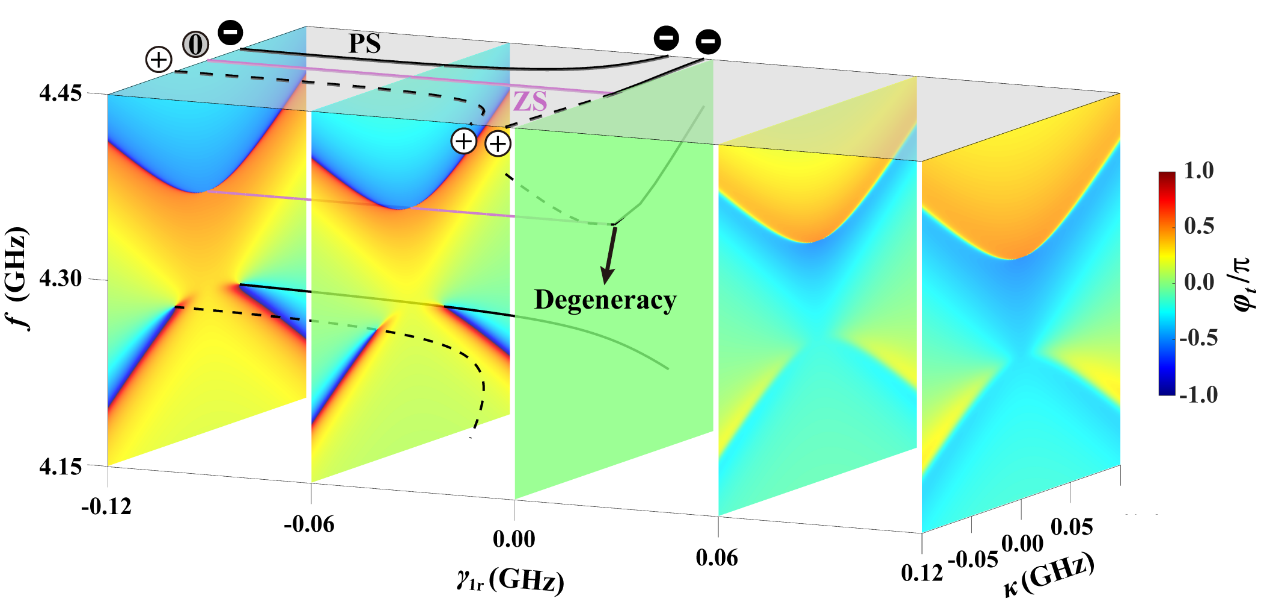


**Figure S13.** **Degeneracy between PS pair and ZS pair.** Susceptibility slices with different $\gamma_{1r}$, and the trajectories of singularities are projected to the top plane. Here $f_{1}=4.344$ GHz, $f_{2}=4.264$ GHz, $\gamma_{1d}=0$ GHz, and $\gamma_{2d}=0.006$ GHz are fixed.

Focus on the transmission $T=\left| t \right|^{2}$ with $t=1-\frac{\gamma_{1r}\left[ i\left( f-f_{2} \right)+\gamma_{2d} \right]}{\left[ i\left( f-f_{1} \right)+\gamma_{1} \right]\left[ i\left( f-f_{2} \right)+\gamma_{2d} \right]+\kappa^{2}}=\frac{\left[ i\left( f-f_{1} \right)+\gamma_{1d} \right]\left[ i\left( f-f_{2} \right)+\gamma_{2d} \right]+\kappa^{2}}{\left[ i\left( f-f_{1} \right)+\gamma_{1} \right]\left[ i\left( f-f_{2} \right)+\gamma_{2d} \right]+\kappa^{2}}$, the numerator and denominator of $t$ exhibit similar analytical forms with $\gamma_{1d}\leftrightarrow\gamma_{1}$. As a result, the ZSs at $\left\{ \begin{matrix} f=(f_{1}\gamma_{2d}+f_{2}\gamma_{1d})/(\gamma_{1d}+\gamma_{2d}) \\ \kappa=\pm\sqrt{-\gamma_{1d}\gamma_{2d}[1+\left( f_{1}-f_{2} \right)^{2}/{(\gamma_{1d}+\gamma_{2d})}^{2}]} \end{matrix} \right.$ (corresponding to the numerator close to zero) and the PSs at $\left\{ \begin{matrix} f=(f_{1}\gamma_{2d}+f_{2}\gamma_{1})/(\gamma_{1}+\gamma_{2d}) \\ \kappa=\pm\sqrt{-\gamma_{1}\gamma_{2d}[1+\left( f_{1}-f_{2} \right)^{2}/{(\gamma_{1}+\gamma_{2d})}^{2}]} \end{matrix} \right.$ (corresponding to the denominator close to zero) inherit the formal similarity. For $\gamma_{1d}=\gamma_{1}$ (namely the radiative loss $\gamma_{1r}=0$) or $\gamma_{2d}=0$ (discussed in **Figure 2** of the main text), the conditions of ZSs and PSs are coincident. As is shown in **Figure S13**, the parameters are set to $f_{1}=4.344$ GHz, $f_{2}=4.264$ GHz, $\gamma_{1d}=0$ GHz, and $\gamma_{2d}=0.006$ GHz. Intriguingly, the pair of ZS carrying opposite topological charges $\nu=\int_{C} \frac{d\varphi_{t}}{2\pi}$ always merges with increasing $\gamma_{1r}$, which is marked by the symbol 0. For $\gamma_{1r}=0$, the ZS pair is degenerate with the PS pair.

However, this jump will arouse a ill-defined but nonphysics $GD=\partial\varphi_{t}/\partial\omega$. Therefore, we employ a translation of $2\pi$ to restore the continuity. And **Figure S14(a)** provides some details of **Figures 2(b, c)**.

*
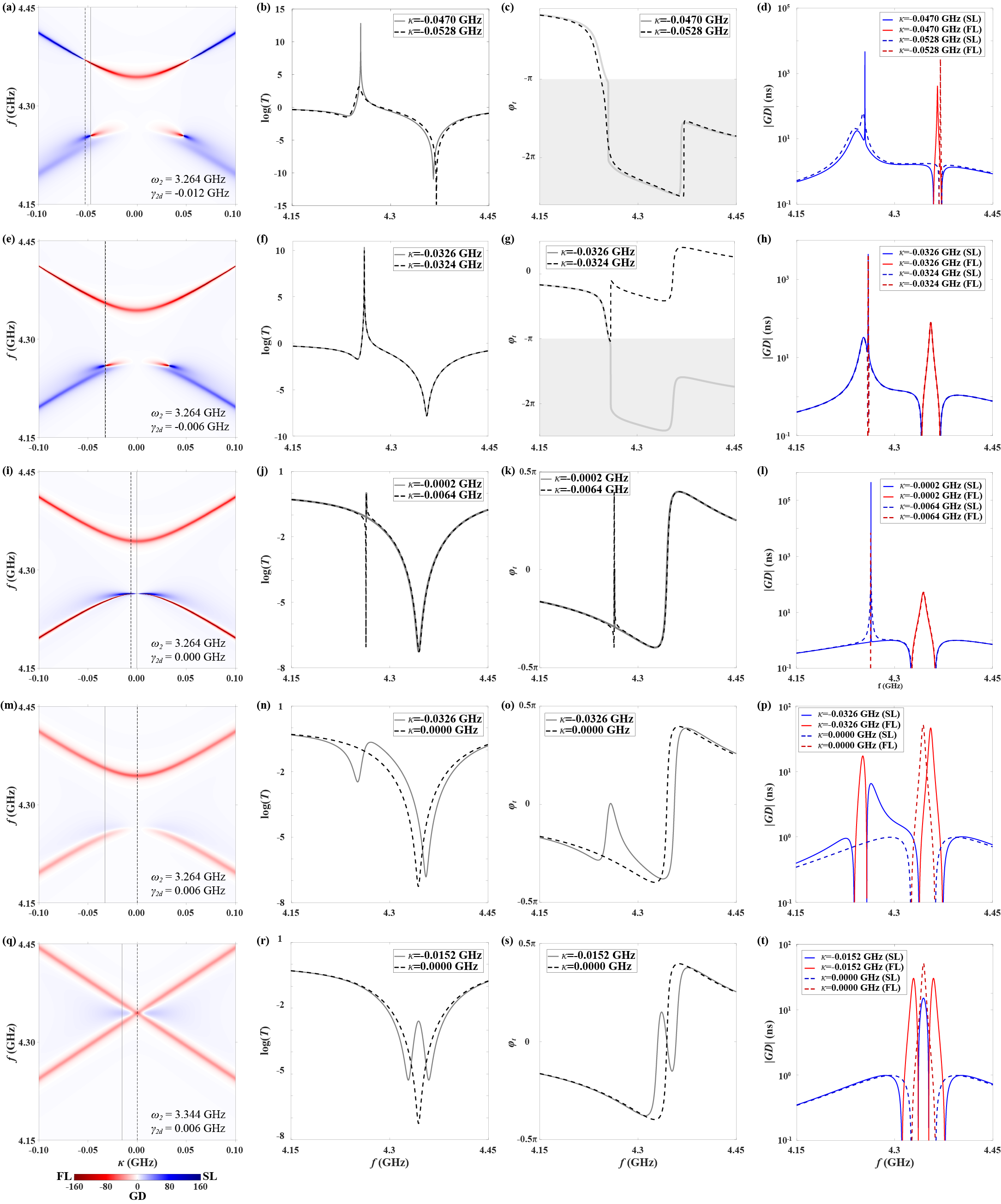
*

**Figure S14.** **Comparison of *GD* in different steps of evolution.** (a) *GD* diagram with $f_{2}=4.264$ GHz and $\gamma_{2d}=-0.012$ GHz. The other parameters are same to Figure 2. The maximum and minimum of *GD* in this region are located at the solid and dashed lines, representing the strongest SL and FL. (b) Transmission $T$, (c) Susceptibility $\varphi_{t}$ after translation, and (d) *GD* along the solid and dashed lines in (a), where blue and red parts correspond to the SL (*GD*>0) and FL (*GD*<0). (e)–(h) Similar to (a)–(d), but for $f_{2}=4.264$ GHz and $\gamma_{2d}=-0.006$ GHz. (i)–(l) Similar to (a)–(d), but for $f_{2}=4.264$ GHz and $\gamma_{2d}=0$ GHz. (m)–(p) Similar to (A)–(D), but for $f_{2}=4.264$ GHz and $\gamma_{2d}=0.006$ GHz. (q)–(t) Similar to (a)–(d), but for $f_{2}=4.344$ GHz and $\gamma_{2d}=0.006$ GHz. The spectral symmetry is improved compared to (m)–(p), which enhances the SL.

# S6. *GD* obtained from the time-domain calculation

To further the slow light property, the *GD* of the signals can be easily obtained from the Keysight (Agilent) ADS (Advanced Design System) simulator [S16]. Considering two signals with $V_{1}(t)=\cos(\omega_{1}t+\varphi_{1})$ and $V_{2}(t)=\cos(\omega_{2}t+\varphi_{2})$, the *GD* can be determined as:

$GD(\omega_{0})=\left. -\frac{d\varphi_{t}(\omega)}{d\omega} \right|_{\omega=\omega_{0}}\approx-\frac{\varphi_{t}(\omega_{1})-\varphi_{t}(\omega_{2})}{\omega_{1}-\omega_{2}}$. (S6. 1)

where $\omega_{1}=\omega_{0}+\Delta\omega/2$ and $\omega_{2}=\omega_{0}-\Delta\omega/2$. The simulation obtained voltage U distributions of $\omega_{1}=2\pi\cdot4.42$ GHz and $\omega_{2}=2\pi\cdot4.39$ GHz in the time domain are shown in **Figures S15(a, b)**, respectively. Compared **Figure S15(a)** with **Figure S15(b)**, the phase difference is $\varphi_{t}(\omega_{1})-\varphi_{t}(\omega_{2})=$ 2.63 rad. According to Equation (S6.1), the *GD* obtained from the time-domain is 4.98 ns, which is well consistent with the measured results in the *GD* spectrum in **Figure 3(c).**

**
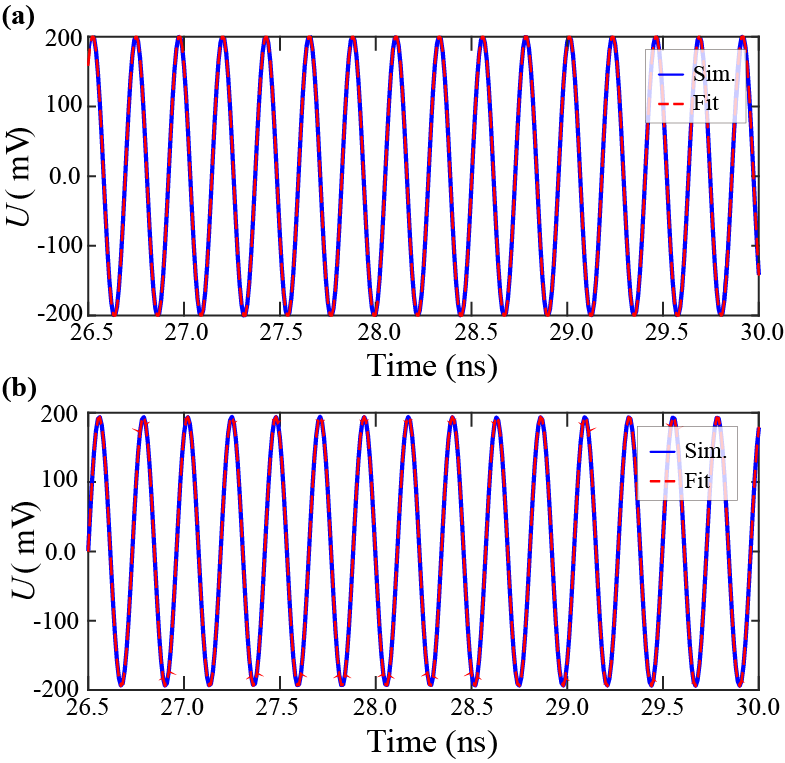
**

**Figure S15**. ***GD* obtained from the time-domain simulations.** (a) The simulation (blue solid line) and fit (red dashed line) obtained sinusoidal signals at the EIT window (4.42 GHz). (b) Similar to (a), but for the sinusoidal signals near the EIT window (4.39 GHz).

# S7. The EIT field distributions of the structure without disorders

In the main text, the measured electric field distributions of the on-chip topological structures are given in **Figure 3**. In the section, the corresponding full-wave simulations are presented for comparison. For the waveguide-based on-chip topological EIT sample with configuration PCB-PCA-PCB, the schematic diagram and the experimental photo are shown in **Figures S16(a, b)**, respectively. Same as the measured electric field distributions in **Figure 3**, the simulated *E_z_* distributions for the bright atom (TBS), dark atom (TDS), and molecule at the EIT window are shown in **Figures S16(c-e)**, respectively.

**
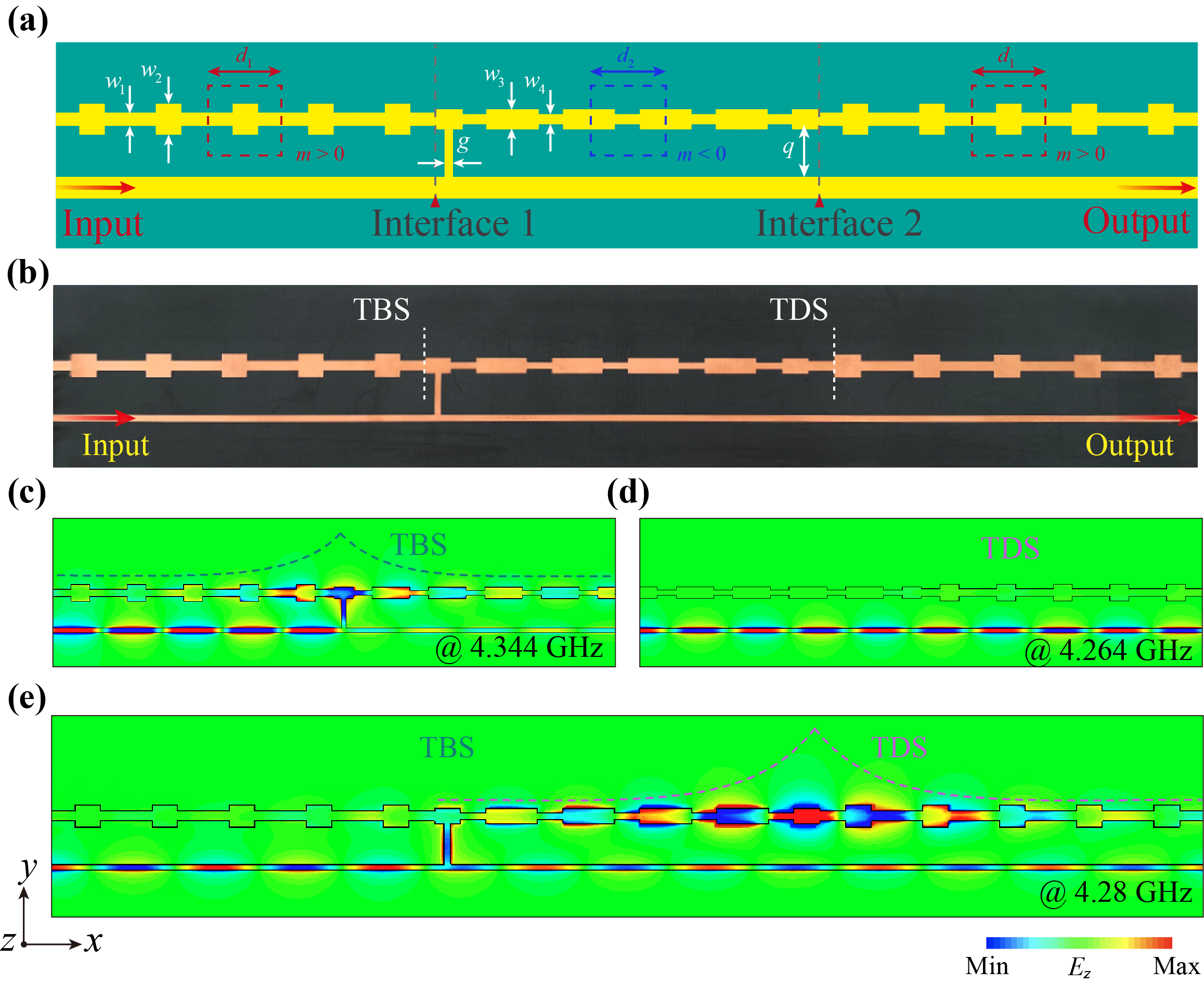
**

**Figure S16.** **Simulated demonstration of the on-chip topological EIT for comparison.** Schematic diagram (a) and the experimental photo (b) of the waveguide-based on-chip topological EIT sample with configuration PC_B_-PC_A_-PC_B_. The bright and dark edge states at interfaces 1 and 2 have been marked by the dashed lines. Full-wave simulated the out-of-plane electric field distribution of on-chip atoms for (c) TBS and (d) TDS. (e) The on-chip topological molecule with TBS and TDS at the EIT window.

Intriguingly, the process of energy transfer from the TBS to the TDS is just like the topological state transfer in acoustic systems [S17] or phononic systems [S18]. Focus on PC_A_ in **Figure S17(a)**, the coupling $\kappa$ between TBS and TDS can be modulated by the period *N*. When N is large enough, TBS will decouple with TDS with $\kappa\to0$, corresponding to the energy localization at the right interface (interface 1), like **Figure S17(b)**. With reducing *N*, the topological edge state can cross PC_A_ gradually to the opposite interface (interface 2) in **Figures S17(b, c)**, coming back to the result of EIT discussed in the main text.

**
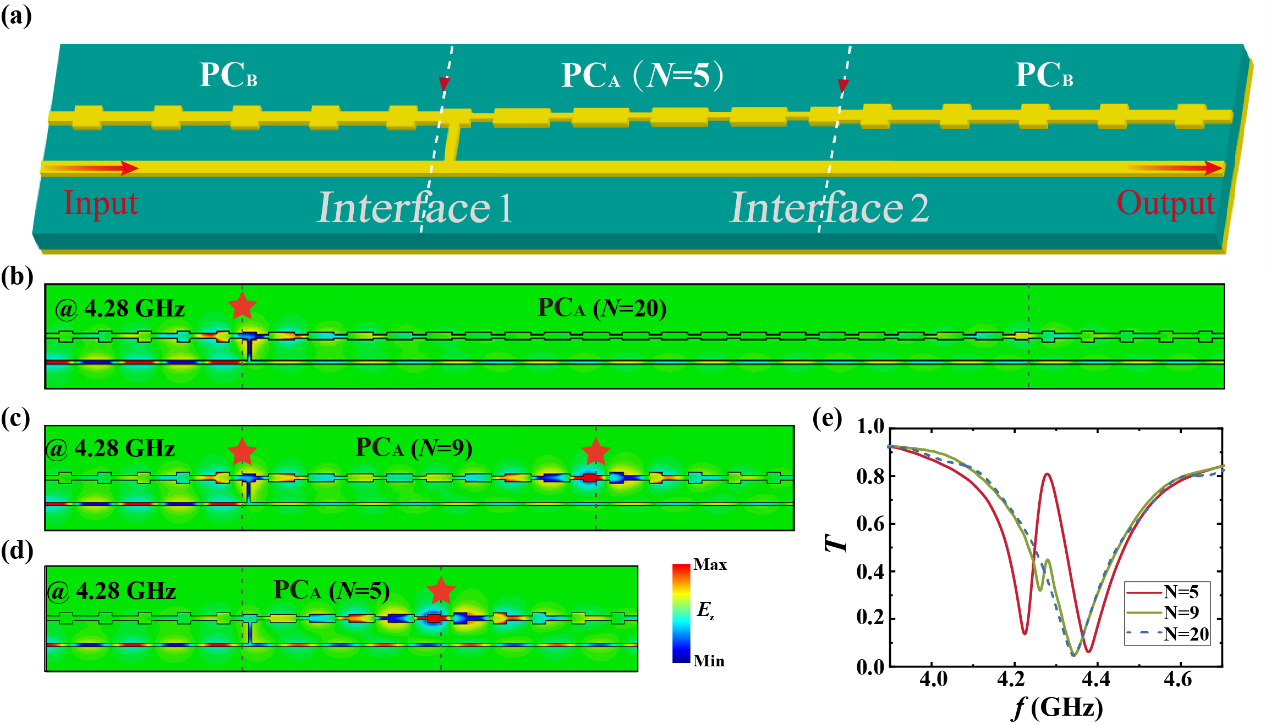
**

**Figure S17.** **Topological state transfer in the topological quantum-interference-like platform.** (a) Schematic of the waveguide-based on-chip topological EIT sample containing a uniform waveguide (lower) and a composite waveguide PC_B_-PC_A_-PC_B_ with varying width (upper). Focus on PC_A_, two topological edge states form at interface 1 and interface 2 marked by dashed lines, which play the respective roles of topological bright state and topological dark state. Full-wave simulated out-of-plane electric field distribution at the frequency of 4.28 GHz, where PC_A_ possesses a period of (b) *N*=20, (c) *N*=9, (d) *N*=5. The red stars mark the position of energy localizations. (e) The related transmission spectra.

**S8. Topological EIT realized by the inversed configuration**

In the main text, the on-chip topological EIT is studied for the sample with configuration PC_B_-PC_A_-PC_B_. In this section, the topological EIT realized by the inversed configuration PC_A_-PC_B_-PC_A_ is uncovered. The effective waveguide-based on-chip topological structure in the transmission-line platform to exhibit the topological EIT is shown in **Figure S18(a)**. The inner waveguide with $m>0$ is sandwiched between two topologically distinguished waveguides with $m<0$, forming two topological interfaces, which are marked by the white dashed lines. The geometric parameters are the same as in the main text. The spectra of the TBS and TDS are shown in the top and bottom panels of **Figure S18(b)**, respectively. Especially, similar to **Figure 3(e)**, the individual topological TBS and TDS are shown by the electric field distributions in the top and bottom panels of **Figure S18(c)**. Moreover, the comparison of simulated and measured EIT transmission (*GD*) spectra are given by the solid and dashed lines in the top (or bottom) panel of **Figure S18(d)**, respectively. The measured results meet well the simulated ones. It should be emphasized that because of the weak dispersion, the *GD* at the topological EIT window in the sample with configuration PC_A_-PC_B_-PC_A_ is lower than that of configuration PC_B_-PC_A_-PC_B_ in the main text. Anyway, regardless of the configuration, topological EIT always exists.

**
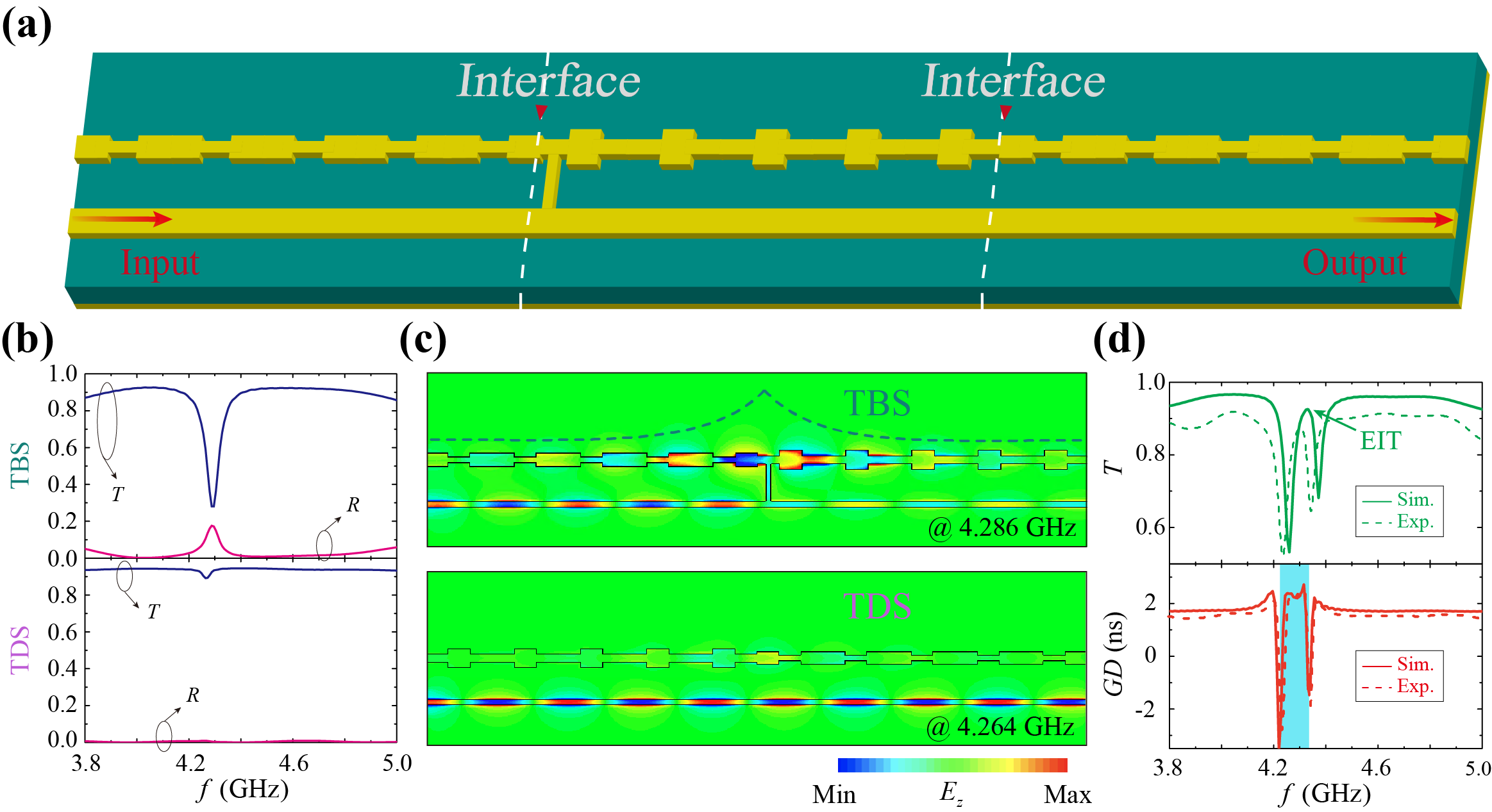
**

**Figure S18.** **Comparison of the on-chip topological EIT in the molecule with configuration PC_A_-PC_B_-PC_A_.** (a) Schematic diagram of the waveguide-based on-chip topological EIT sample containing a simple waveguide with consistent width (lower) and a composite waveguide with varying width (upper). (b) Simulated spectra of the TBS and TDS, which are shown in the top and bottom panels, respectively. (c) Full-wave simulated the out-of-plane electric field distribution of on-chip topological bright (top) and dark (bottom) atoms. (d) The transmission (top) and *GD* (bottom) spectra of the sample. The simulated and measured spectra are marked by the solid lines and dashed lines, respectively.

The on-chip topological EIT is further observed in **Figure S19**. The experimental sample of the sample with configuration PC_A_-PC_B_-PC_A_ is shown in **Figure S19(a)**. At the EIT window, the simulated *E_z_* and measured |*E_z_*| distributions of the EIT window for the on-chip topological molecule with bright and dark atoms are shown in the top and bottom panels of **Figure S19(b)**, respectively. It can be clearly seen that the electric field will transform from the bright atom to the dark atom at the EIT window, indicating the on-chip topological EIT phenomenon in the configuration PC_A_-PC_B_-PC_A_.

**
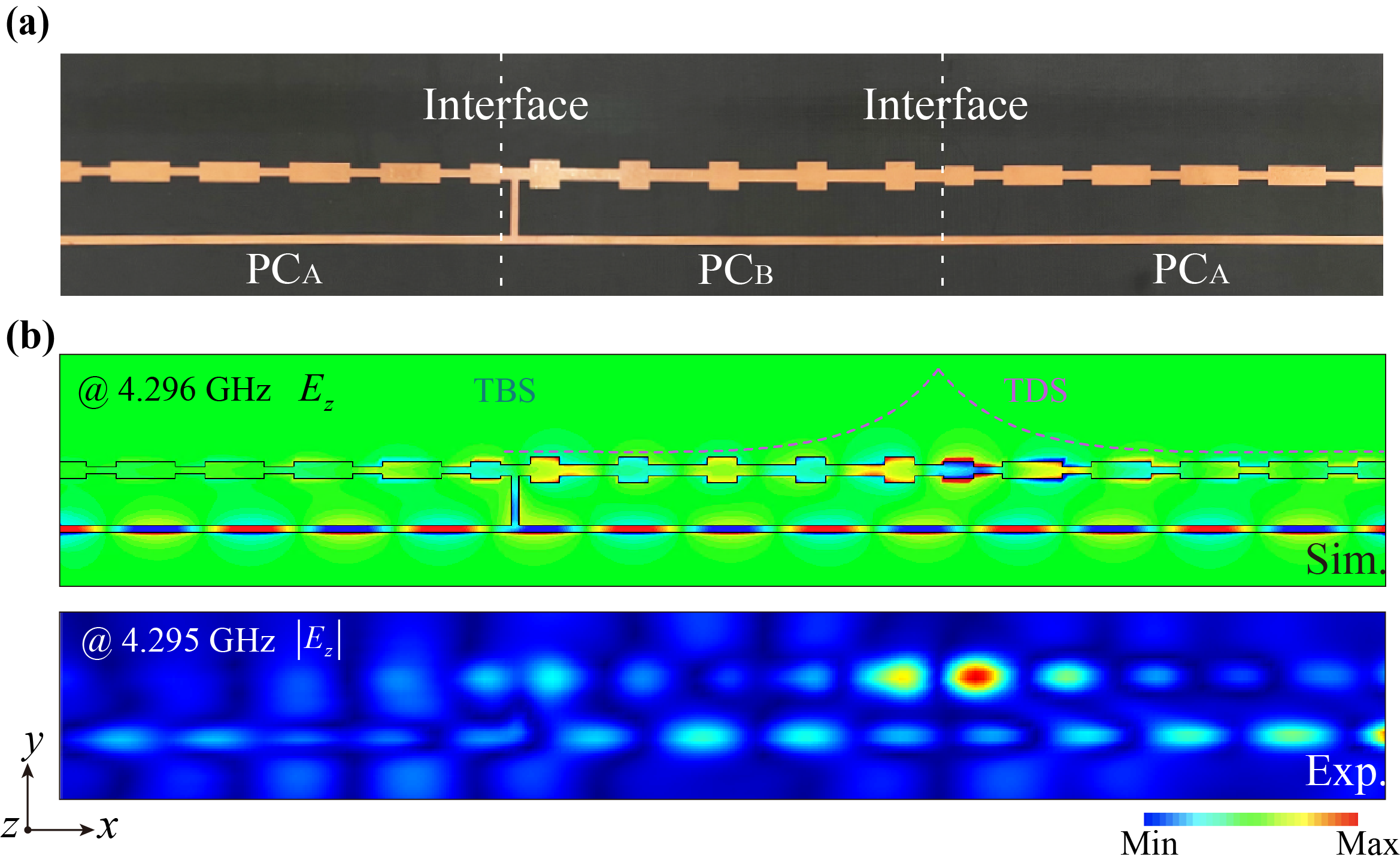
**

**Figure S19.** **Comparison of the electric field distributions of topological EIT in the molecule with configuration PC_A_-PC_B_-PC_A_.** (a) Experimental sample of the topological molecule with configuration PCA-PCB-PCA. (b) Simulated *E_z_* and measured |*E_z_*| distributions of the EIT window for the on-chip topological molecule with bright and dark atoms.

# S9. The topological EIT for different disorder configurations

In the main text, we have demonstrated that the topological EIT realized by edge states is robust against the structure disorder (i.e., type-I disordered configuration). In this section, to further verify the topological protection, the other three disordered configurations are considered. The specific implementation of four types of disorder for $\eta=1$ mm is shown in Table S1. The simulated transmission and group delay (*GD*) spectra are shown in the top panels of **Figures S20(c, d)**, respectively. It is clear that the EIT window and slow light effect are indeed perfectly preserved.

| Disorder  Number | **Type-I** | **Type-II** | **Type-III** | **Type-IV** |
| --- | --- | --- | --- | --- |
| ${\Delta w}_{1}$ | 0.45 | -0.62 | -0.21 | -0.57 |
| ${\Delta w}_{2}$ | 0.01 | -0.62 | -0.87 | -0.12 |
| ${\Delta w}_{3}$ | 0.98 | 0.53 | 0.59 | -0.57 |
| ${\Delta w}_{4}$ | 0.65 | -0.17 | 0.04 | -0.42 |
| ${\Delta w}_{5}$ | -0.71 | 0.37 | 0.29 | 0.98 |
| ${\Delta w}_{6}$ | 0.76 | 0.46 | -0.89 | -0.75 |
| ${\Delta w}_{7}$ | 0.18 | -0.64 | -0.64 | -0.90 |
| ${\Delta w}_{8}$ | -0.31 | 0.06 | 0.23 | -0.37 |
| ${\Delta w}_{9}$ | -0.48 | -0.14 | -0.92 | 0.64 |
| ${\Delta w}_{10}$ | 0.64 | -0.71 | 0.46 | -0.68 |
| ${\Delta w}_{11}$ | 0.32 | 0.90 | 0.53 | -0.01 |
| ${\Delta w}_{12}$ | -0.98 | -0.01 | -0.78 | -0.67 |
| ${\Delta w}_{13}$ | 0.09 | 0.76 | -0.96 | -0.06 |
| ${\Delta w}_{14}$ | 0.75 | 0.75 | 0.07 | 0.60 |
| ${\Delta w}_{15}$ | 0.45 | 0.35 | 0.71 | 0.65 |
| ${\Delta w}_{16}$ | 0.20 | 0.50 | 0.50 | 0.53 |
| ${\Delta w}_{17}$ | 0.06 | -0.54 | 0.48 | 0.59 |
| ${\Delta w}_{18}$ | 0.02 | -0.89 | 0.48 | -0.25 |
| ${\Delta w}_{19}$ | 0.12 | 0.14 | 0.29 | -0.87 |
| ${\Delta w}_{20}$ | 0.92 | -0.42 | 0.68 | 0.50 |
| ${\Delta w}_{21}$ | 0.21 | 0.48 | 0.10 | -0.28 |
| ${\Delta w}_{22}$ | -0.95 | -0.15 | -0.14 | -0.31 |
| ${\Delta w}_{23}$ | 0.71 | -0.85 | -0.50 | -0.98 |
| ${\Delta w}_{24}$ | -0.23 | -0.34 | 0.65 | -0.75 |
| ${\Delta w}_{25}$ | -0.75 | 0.09 | -0.25 | -0.98 |
| ${\Delta w}_{26}$ | 0.70 | -0.60 | 0.20 | 0.56 |
| ${\Delta w}_{27}$ | 0.31 | 0.01 | -0.92 | -0.18 |
| ${\Delta w}_{28}$ | -0.67 | -0.01 | 0.04 | 0.01 |
| ${\Delta w}_{29}$ | -0.17 | -0.57 | -0.01 | -0.85 |
| ${\Delta w}_{30}$ | -0.09 | 0.37 | -0.04 | -0.71 |
| ${\Delta w}_{31}$ | 0.46 | 0.81 | -0.09 | -0.21 |
| ${\Delta w}_{32}$ | -0.20 | -0.65 | 0.81 | 0.98 |
| ${\Delta w}_{33}$ | -0.92 | 0.40 | 0.57 | -0.79 |
| ${\Delta w}_{34}$ | 0.75 | -0.35 | -0.95 | 0.70 |
| ${\Delta w}_{35}$ | -0.20 | 0.89 | -0.43 | -0.79 |
| ${\Delta w}_{36}$ | 0.89 | 0.23 | 0.67 | 0.35 |
| ${\Delta w}_{37}$ | 0.76 | 0.25 | -0.18 | -0.15 |
| ${\Delta w}_{38}$ | 0.67 | 0.29 | 0.48 | -0.12 |
| ${\Delta w}_{39}$ | 0.64 | -0.62 | 0.93 | -0.82 |
| ${\Delta w}_{40}$ | -0.18 | 0.39 | 0.60 | 0.60 |
| ${\Delta w}_{41}$ | 0.62 | 0.40 | -0.12 | -0.53 |
| ${\Delta w}_{42}$ | 0.89 | 0.96 | -0.79 | 0.56 |
| ${\Delta w}_{43}$ | 0.73 | 0.75 | -0.93 | -0.45 |
| ${\Delta w}_{44}$ | 0.96 | -0.39 | 0.64 | 0.32 |
| ${\Delta w}_{45}$ | 0.95 | 0.29 | 0.17 | 0.01 |

Table S1. Four types of disorder configurations.


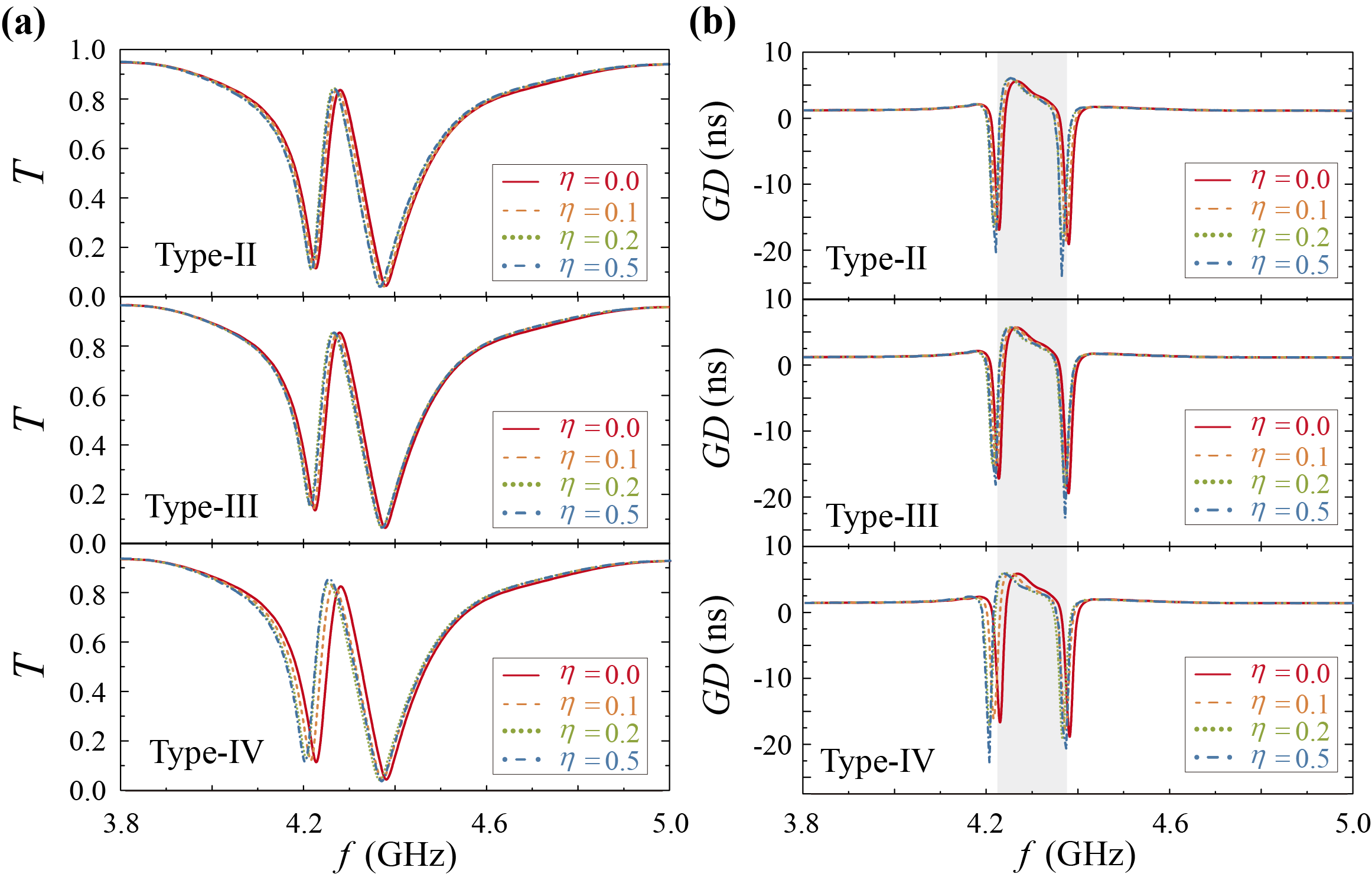


**Figure S20. The robustness of on-chip topological EIT for different disordered configurations.** (a) Simulated transmission spectra of the type-II (top), type-III (middle), and type-IV (bottom) disordered structures. (b) Similar to (A), but for the *GD* spectra.

# S10. The EIT field distributions of structures with different width disorder strength

In the main text, the width disorder robustness of on-chip topological EIT is experimentally demonstrated in **Figure 4**. In **Figure S21**, the simulated electric field distributions of the on-chip topological molecule with disorder strength $\eta=$0.1 mm, 0.2 mm, and 0.5 mm are shown in the top, middle, and bottom panels, respectively. The experimental demonstration of the disorder robustness of on-chip topological EIT in the main text meets well with simulated ones in **Figure S21**.

**
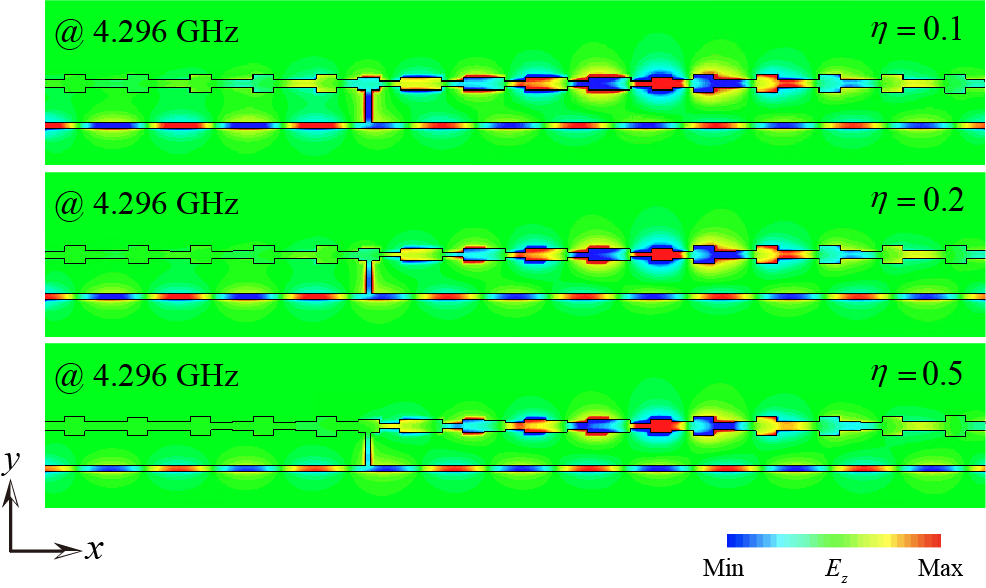
**

**Figure S21.** **Simulation demonstrated the robustness of on-chip topological EIT.** Full-wave simulated the electric field distributions of on-chip topological molecules with different disorder strengths: (top) $\eta\boldsymbol{=}$0.1 mm; (middle) $\eta\boldsymbol{=}$0.2 mm; (bottom) $\eta\boldsymbol{=}$0.5 mm.

# S11. Robustness of the topological EIT for the structural bending

The topological EIT realized by the sample with configuration PC_A_-PC_B_-PC_A_ is also immune to structural bending. **Figure S22(a)** shows the experimental photo of the waveguide-based on-chip topological EIT sample with one bend. The comparison of simulated transmission spectra with and without bends is shown in **Figure S22(b)**. Moreover, similar to the simulated transmission spectra, the comparison of measured ones is shown in **Figure S22(c)**. From **Figures S22(b, c)**, it can be found that the EIT window is almost unaffected by structural bending, except for a slight frequency offset. The simulated electric field distribution of the sample with one bend is shown in **Figure S22(d)**. Same as **Figure S22(d)**, the measured electric field distribution with and without phase are shown in **Figures S22(e, f)**, respectively.

**
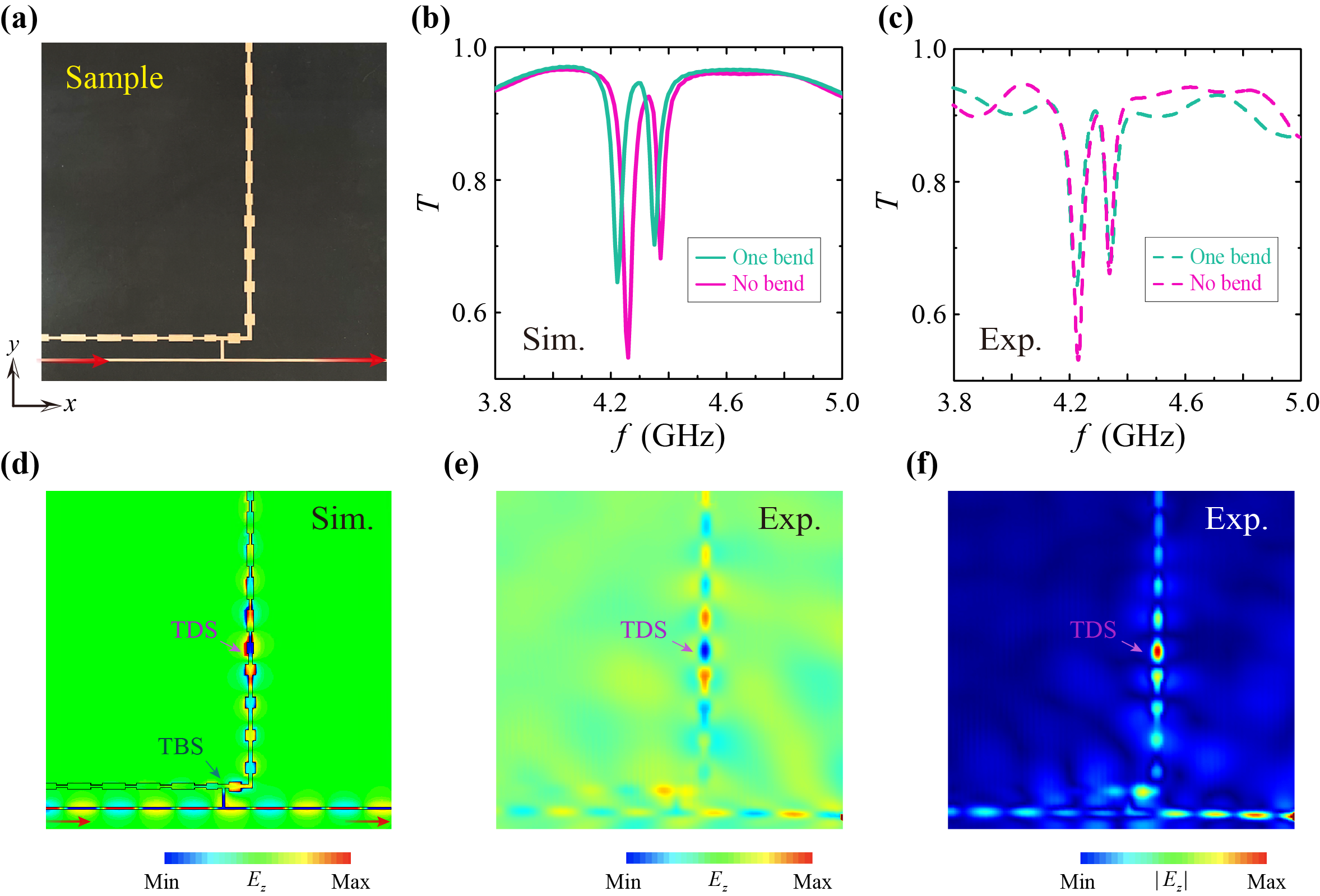
**

**Figure S22. Comparison of the robustness of on-chip topological EIT for the sample with one bend.** (a) Experimental photo of the waveguide-based on-chip topological EIT sample with configuration PC_A_-PC_B_-PC_A_. Comparison of the simulated (b) and measured (c) transmission spectra of the sample with (green lines) and without (pink lines) bend. (d) Simulated electric field distribution of on-chip topological molecule with one bend. Same as (d), (e), and (f) correspond to the measured electric field distribution with and without phase, respectively.

Furthermore, considering two bends, as shown by the experimental sample in **Figure S23(a)**, the topological protection of EIT established by edge states is studied. Similar to **Figures S22(b, c)**, the comparison of simulated and measured transmission spectra for the samples with two bends is shown in **Figures S23(b, c)**, respectively. The simulated electric field distribution is shown in **Figure S23(d)**, and the electric field distribution is almost undisturbed. Moreover, the corresponding measured electric field distribution with and without phase are shown in **Figures S23(e, f)**, respectively. The measured results meet well with the simulated ones. Therefore, from **Figures S22** and **S23** the topological protection of the topological EIT in the sample with configuration PC_A_-PC_B_-PC_A_ is clearly demonstrated.


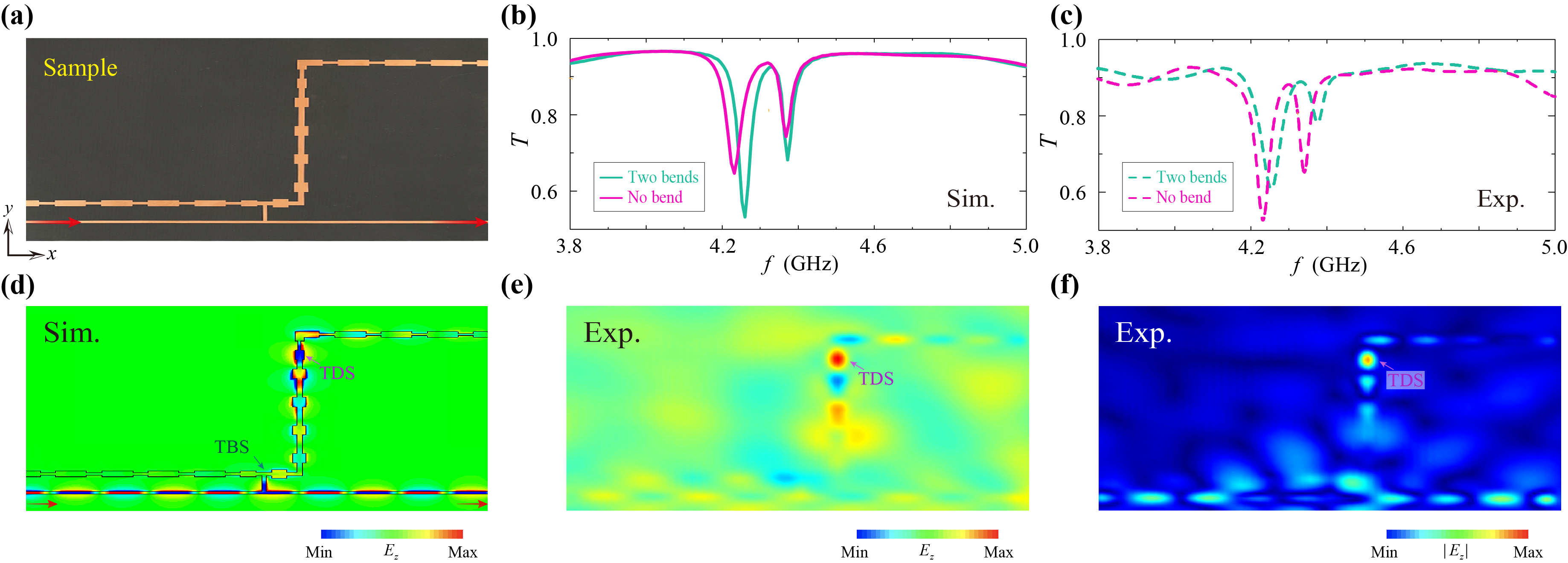


**Figure S23. Comparison of the robustness of on-chip topological EIT for the sample with two bends.** (a) Experimental photo of the waveguide-based on-chip topological EIT sample with configuration PC_A_-PC_B_-PC_A_. Comparison of the simulated (b) and measured (c) transmission spectra of the sample with (green lines) and without (pink lines) bend. (d) Simulated electric field distribution of on-chip topological molecule with one bend. Same as (d), (e), and (f) correspond to the measured electric field distribution with and without phase, respectively.

As shown in **Figures 4(g)**, **S22(b)**, and **S23(b)**, structural bending induces a frequency shift. This arises from changes in the propagation constant and local refractive index caused by the waveguide deformation, leading to a shift of approximately 0.06 GHz. However, this shift is significantly smaller than the EIT bandwidth. Nevertheless, due to the topological protection mechanism, the EIT phenomenon remains stable, demonstrating the system’s robust performance under structural variations.

Through experiments, we have effectively verified the robustness of the topological EIT when structural bending is introduced. Here, we present the simulated GD spectra in **Figure S24(a)** and experimental GD spectra in **Figure S24(b)** corresponding to the structure in **Figure 4(f)** of the main text. Both the simulated and experimental results show that the system is able to maintain the slow light effect even after undergoing deformation. These results further demonstrate the topological protection characteristics of the system, which continues to exhibit strong stability and robustness under structural bending deformations.

*
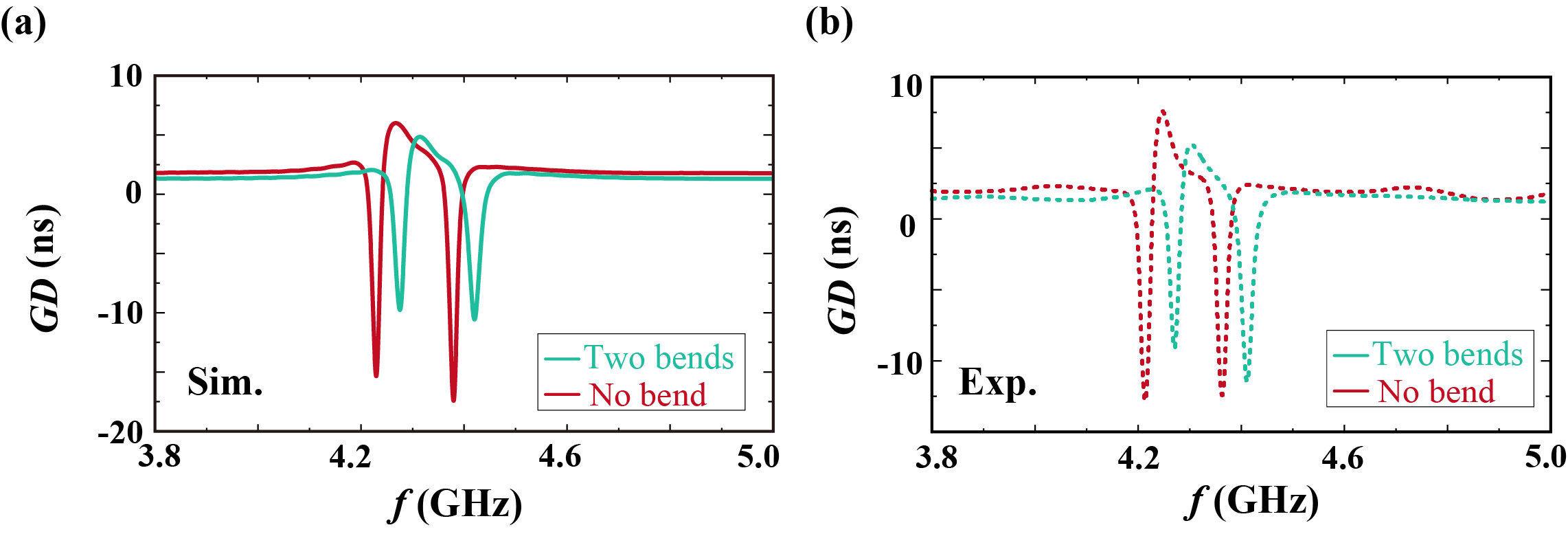
***Figure S24.** **Comparison of the *GD* for the on-chip topological EIT in the sample with two bends and the sample without bending.** Simulated (a) and measured (b) *GD* spectra for the sample with (green line) and without (red line) bends.

The relatively strong electric field observed in the dielectric region during the experiment [**Figures 4(h), S22(e)**, and **S23(e)**] is due to the combined effect of various experimental and physical factors. Due to the limited spatial resolution of the near-field probe (approximately 5 cm in length), the measurement not only captures the local field at the waveguide interface, but also inevitably receives radiation fields from metal microstrip lines and stray fields caused by substrate edge diffraction—these effects are suppressed in simulations under ideal boundary conditions, but are difficult to completely avoid in practical experiments. In addition, there are slight unevenness in the foam substrate, background noise (such as cable radiation) in the instrument system, and processing defects such as rough edges or microcracks in the device structure, which may also lead to energy leakage into the radiation mode, thus enhancing the electric field strength in the environmental medium. However, the transmission spectrum shown in **Figure 4(d)** and the group delay in **Figure 4(g)** were not affected, indicating that the topological EIT effect has good robustness and the background electric field does not affect the main physical mechanism.

# S12. Robustness of the topological EIT for the structural deformation

The physical model of on-chip topological EIT proposed in this work is generalized. In this part, the robustness of the topological EIT for the structural deformation is also demonstrated. **Figure S25(a)** shows the schematic diagram of the waveguide-based on-chip topological EIT sample containing a simple waveguide with consistent width (lower) and two same composite waveguides with varying widths (middle and upper). The simulated transmission and *GD* spectra of the transformed structure are shown in the top and bottom panels of **Figure S25(b)**, respectively. Because of the larger radiative loss in this configuration, the EIT window shown in the transmission spectra is wider than the untransformed structures with configurations PC_B_-PC_A_-PC_B_ and PC_A_-PC_B_-PC_A_ in **Figures 3** and **S17**, respectively. Nevertheless, the topological window also exists. The corresponding electric field distribution of the EIT window in this transformed structure is shown in Figure S19(c). The energy is also strongly confined to the dark atom instead of the bright atom because of the destructive interference.

**
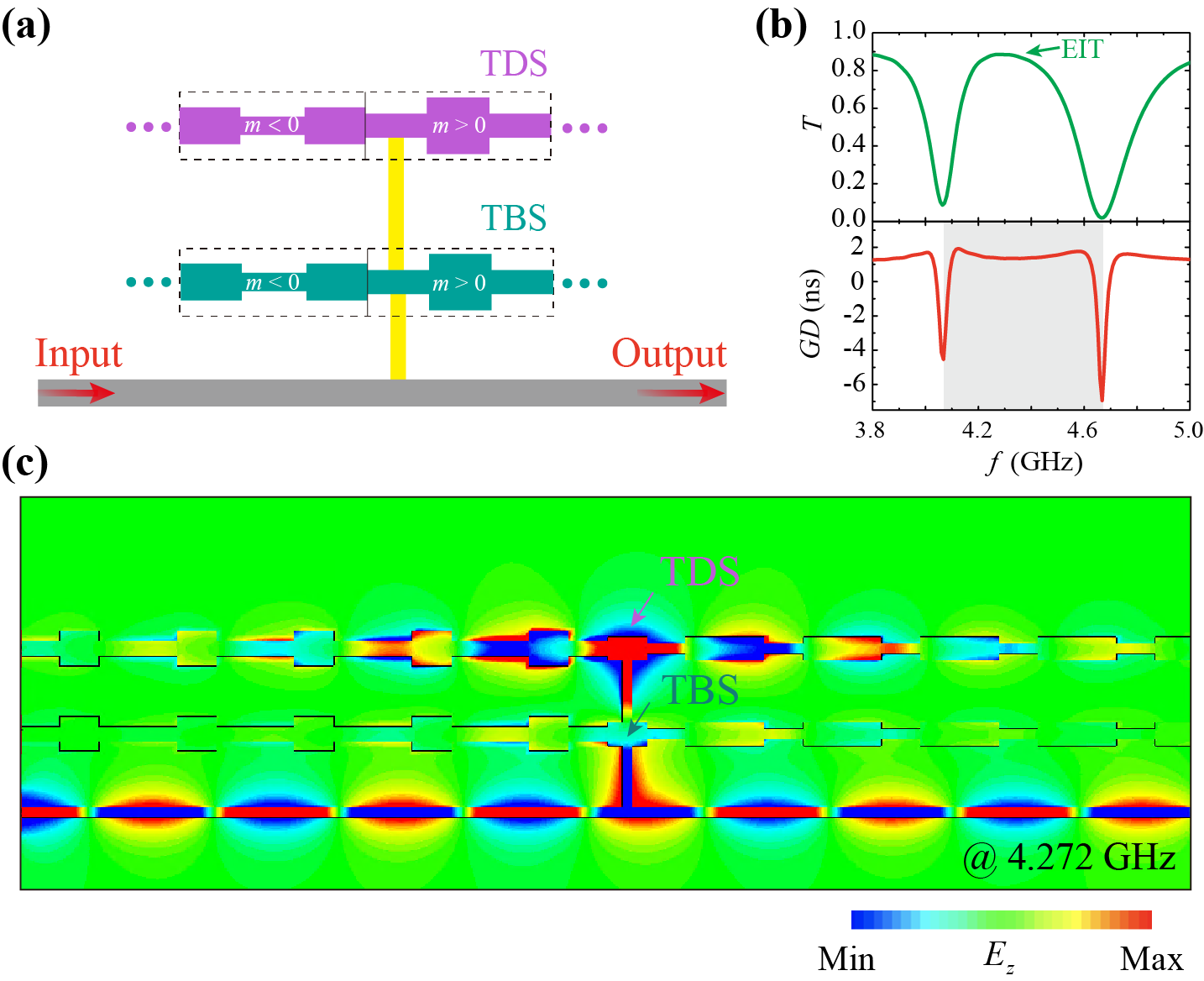
**

**Figure S25.** **Comparison of the on-chip topological EIT in the structural deformed molecule with the configuration of double PC_A_-PC_B_.** (a) Schematic diagram of the waveguide-based on-chip topological EIT sample containing a simple waveguide with consistent width (lower) and two same composite waveguides with varying width (middle and upper). (b) The simulated transmission (top) and *GD* (bottom) spectra of the structure. (c) Simulated *E_z_* distribution of the EIT window for the on-chip topological molecule with bright and dark atoms.

**References**

[S1] A. Yariv and P. Yeh, Optical Waves in Crystals: Propagation and Control of Laser Radiation Wiley, New York **1984**.

[S2] M. Xiao, Z. Q. Zhang, and C. T. Chan, Surface Impedance and Bulk Band Geometric Phases in One-Dimensional Systems, *Phys. Rev. X* **2014**, 4, 021017.

[S3] Zak, J. Symmetry criterion for surface states in solids. *Phys. Rev. B* **1985**, 32, 2218.

[S4] M. Xiao, K. Ding, Z. Y. Yang, P. Sheng, Z. Q. Zhang, and C. T. Chan, Geometric phase and band inversion in periodic acoustic systems, *Nat. Phys*. **2015**, 11, 240.

[S5] S. Q. Shen, Topological Insulators: Dirac Equation in Condensed Matters (Springer Science & Business Media, **2012**).

[S6] W. Tan, Y. Sun, H. Chen, and S. Q. Shen, Photonic simulation of topological excitations in metamaterials, *Sci. Rep*. **2014**, 4, 3842.

[S7] X. Shi, C. H. Xue, H. T. Jiang, and H. Chen, Topological description for gaps of one dimensional symmetric all-dielectric photonic crystals, *Opt. Express* **2016**, 24, 18580.

[S8] Q. S. Huang, Z. W. Guo, J. T. Feng, C. Y. Yu, H. T. Jiang, Z. Zhang, Z. S. Wang, and H. Chen, Observation of a Topological Edge State in the X-ray Band, *Laser Photon. Rev*. **2019**, 13, 1800339.

[S9] Z. W. Guo, Y. Q. Wang, S. L. Ke, X. Q. Su, J. Ren, and H. Chen, One-dimensional photonic topological structures composed of meta-atoms and their applications, *Adv. Phys. Res*. **2023**, 2300125.

[S10] M. Hu, K. Ding, T. Qiao, X. Jiang, Q. Wang, S. N. Zhu, and H. Liu, Realization of photonic charge-2 Dirac point by engineering super-modes in topological superlattices, *Commun. Phys*. **2020**, 3, 130.

[S11] T. Qiao, M. Hu, X. Jiang, Q. Wang, S. N. Zhu, and H. Liu, Generation and Tunability of Supermodes in Tamm Plasmon Topological Superlattices, *ACS Photon*. **2021**, 8, 2095.

[S12] C. Wang, W. R. Sweeney, A. D. Stone, and L. Yang, Coherent perfect absorption at an exceptional point, *Science* **2021**, 373, 1261.

[S13] M. Milićević, G. Montambaux, T. Ozawa, O. Jamadi, B. Real, I. Sagnes, A. Lemaître, L. Le Gratiet, A. Harouri, J. Bloch, A. Amo, Type-III and tilted Dirac cones emerging from flat bands in photonic orbital graphene. Phys. Rev. X 2019, 9, 031010.

[S14] Y. Sun, W. Tan, H. Q. Li, J. Li, and H. Chen, Experimental demonstration of a coherent perfect absorber with PT phase transition, *Phys. Rev. Lett*. **2014**, 112, 143903.

[S15] Y. R. Jian, Y. Q. Wang, Z. W. Guo, S. Y. Hu, B. T. Wu, Y. P. Yang, and H. Chen, External excitation enabled chirality reversal of exceptional points in an effective anti-PT-symmetric non-Hermitian system, *Appl. Phys. Lett*. **2023**, 123, 141702.

[S16] J. Ran, Y. W. Zhang, X. D. Chen, K. Fang, J. F. Zhao, Y. Sun, and H. Chen, Realizing Tunable Inverse and Normal Doppler Shifts in Reconfigurable RF Metamaterials, *Sci. Rep*. **2015**, 5, 11659.

[S17] Z.-G. Chen, W. Y. Tang, R.-Y. Zhang, Z. X. Chen, G. C. Ma, Landau-Zener transition in the dynamic transfer of acoustic topological states. *Phys. Rev. Lett.* **2021**, 126, 054301.

[S18] T. Tian, Y. C. Zhang, L. Zhang, L. H. Wu, S. C. Lin, J. W. Zhou, C.-K. Duan, J.-H. Jiang, J. F. Du, Experimental realization of nonreciprocal adiabatic transfer of phonons in a dynamically modulated nanomechanical topological insulator. *Phys. Rev. Lett.* **2022**, 129, 215901.
